# SERS/TERS Characterization of New Potential Therapeutics: The Influence of Positional Isomerism, Interface Type, Oxidation State of Copper, and Incubation Time on Adsorption on the Surface of Copper(I) and (II) Oxide Nanoparticles

**DOI:** 10.1021/acs.jmedchem.2c00031

**Published:** 2022-03-01

**Authors:** Edyta Proniewicz, Tomasz K. Olszewski

**Affiliations:** † Faculty of Foundry Engineering, AGH University of Science and Technology, ul. Reymonta 23, 30-059 Kraków, Poland; ‡ Department of Chemistry, School of Science and Technology, Kwansei Gakuin University, Gakuen 2-1, Sanda, Hyogo 669-137, Japan; § Faculty of Chemistry, Wroclaw University of Science and Technology, Wybrzeże Wyspiańskiego 27, 50-370 Wroclaw, Poland

## Abstract

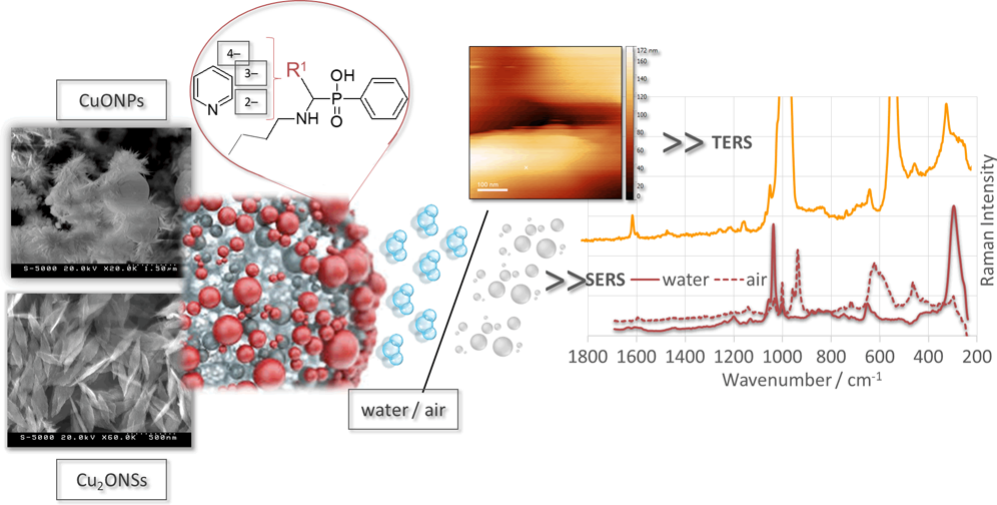

The aim of this study was to investigate how the oxidation state of copper (Cu(I) vs Cu(II)), the nature of the interface (solid/aqueous vs solid/air), positional isomerism, and incubation time affect the functionalization of the surface of copper oxide nanostructures by [(butylamino)(pyridine)methyl]phenylphosphinic acid (PyPA). For this purpose, 2-, 3-, and 4-isomers of PyPA and the nanostructures were synthesized. The nanostructure were characterized by UV-visible spectroscopy (UV–vis), scanning electron microscopy (SEM), Raman spectroscopy (RS), and X-ray diffraction (XRD) analysis, which proved the formation of spherical Cu_2_O nanoparticles (Cu_2_ONPs; 1500–600 nm) and leaf-like CuO nanostructures (CuONSs; 80–180/400–700 nm, width/length). PyPA isomers were deposited on the surface of NSs, and adsorption was investigated by surface-enhanced Raman scattering (SERS) and tip-enhanced Raman scattering (TERS). The changes of adsorption on the surface of copper oxide NSs caused by the above-mentioned factors were described and the enhancement factor on this substrate was calculated.

## Introduction

The electronic and optical properties of metals depend on their surface structures. Nanostructures of metals (MNSs) are characterized by a high surface-to-volume ratio, a spectral shift in fluorescence emission, extremely low energy concentration efficiency, and the ability to localize optical fields at the nanoscale.^1−3^ In addition, the interaction of the MNS surface with the surrounding medium is strong enough to prevail in density differences.^4^ Magnetism, quantum confinement, enhanced toxic properties, lower melting temperature, and different light absorption compared to solid metals are also size-dependent properties of MNS.^4,5^ Therefore, the methods for synthesizing MNSs have been improved to achieve a large active surface area^6^ and nanoscale quantum confinement effects^7^ in a medium free of surfactants to avoid any interference with the latter.^8,9^ Among the various synthesized MNS, metal oxide nanostructures (MONSs) synthesized by anodic electrochemical dissolution of metals are particularly important due to their purity and unique electronic,^10^ electrochemical,^11^ catalytic,^12^ and magnetic^13^ properties.

Among metal oxides, copper oxides occupy an important place. Copper(I) oxide (Cu_2_O) and copper(II) oxide (CuO), the most common initial corrosion product formed during the slow oxidation of metallic copper in contact with the atmosphere, play a special role because they are nontoxic, cheap, and have unique optoelectronic properties, owing to which they have a broad range of technological and medical applications. Applications range from the production of electrodes, sensors, efficient photocatalysts driven by visible light,^14^ solar energy conversion systems,^15^ optoelectronics, lithium-ion batteries,^16^ and superconductors^17^ to cosmetics and textile components,^18^ antiseptics, and bactericides used in daily life or to eliminate pathogens in the aquatic environment.^19^

CuO and Cu_2_O are p-type semiconductors (*E*_g_ = 1.2–1.8 and 2.17 eV, respectively). Cu_2_O belongs to the space group *Pn*3*m* and crystallizes in a cubic structure with a lattice constant of *a* = 4.2696 Å, formed by a body-centered cubic (bcc) arrangement of oxygen atoms with metal atoms between two successive oxygen layers so that each oxygen atom is surrounded by a tetrahedron of copper atoms (a centered cubic (fcc) sublattice) and each metal atom has two coordinates forming linear CuO_2_ units.^20^ The length of the Cu–O bonds in these units is 1.849 Å, which is smaller than in copper(II) oxide.^20^ CuO belongs to the point symmetry group 2/*m* and crystallizes in a monoclinic structure,^21^ in which the environment of the Cu(II) ions is strongly distorted by the strong Jahn–Teller effect. The lengths of the Cu–O bonds in square planar groups are larger than those in Cu_2_O and are 1.88 and 1.96 Å.^22^ The length of the two perpendiculars to the planar Cu–O bonds is much larger, which precludes octahedral coordination.

Due to the wide use of metal oxide nanoparticles, their release into the environment is inevitable. Some MONSs, such as copper oxide NSs, are susceptible to dissolution in environmental systems depending on the size of these nanoparticles, which is of particular concern in the plant rhizosphere.^22,23^ This can lead to the simultaneous release of Cu ions from the surface, increasing the bioavailability of the surface layer and potentially causing negative responses in environmental systems (e.g., inhibition of plant growth) even at subtoxic concentrations of metal ions.^24^ However, even less is known about the negative effects of copper oxide NPs on animal organisms. To this end, the influence of morphology (shape and size) and the concentration of copper oxide NPs on their antimicrobial activity have been studied.^25−27^ For example, it was found that the antimicrobial activity (via the redox cycle between Cu(I) and Cu(II)^28^) of copper oxide NPs against pathogenic microorganisms depends on the oxidation state of copper (Cu(I) ions have a higher potency than Cu(II) ions), pH, size (<100 nm; a decrease in size leads to an increase in antimicrobial activity), and morphology.^29^ Cu(II) ions in bacterial cells are thought to be reduced by sulfhydryl to Cu(I) ions, which are responsible for causing oxidative stress.^30^ The smallest Cu_2_ONPs showed the highest antimicrobial efficiency at intermediate concentrations compared to low (5 mg/mL) or high (200 mg/mL) concentrations.^22^ Cu_2_ONPs were also shown to induce apoptosis in HeLa and melanoma cells at lower concentrations than in normal human and mouse cell lines.^31^ The selective cytotoxicity of copper oxide NPs in cancer cells was also described.^32^ The endoplasmic reticulum stress-induced mechanism on copper oxide NPs has been found to play a role in the apoptosis of renal cancer cells.^25^ Copper oxide NPs also generate reactive oxygen species (ROS) and damage mitochondrial membranes.^33^ The photodynamic activity of copper oxide NPs, resulting from their ability to generate ROS when excited by light, can be used in photodynamic therapy for cancer treatment. However, the tumor-specific toxicity of copper oxide NPs to cancer cells is not yet clear. For example, the octahedral Cu_2_O nanocrystals were shown to have higher photodynamic activity (greater killing capacity) compared to Cu_2_ONPs with cubic and hexagonal shapes.^26^ Moreover, the results show that the viability of cancer cells decreases correspondingly with increasing concentration of Cu_2_ONPs after green laser irradiation.^26^ Other results confirm the positive cytotoxic effect of copper oxide NSs on cancer cell lines by reducing angiogenesis and inducing apoptosis.^34,35^ Other studies have developed nonenzymatic glucose sensors based on copper oxide NSs.^36,37^ It has been also shown that the presence of copper accelerates the process of glucose oxidation and increases the stability of the sensor itself.

As mentioned above, the antibacterial and antifungal properties of copper oxide NSs and their potential use (e.g., for cancer imaging and therapy (shortening the relaxation time of the magnetic spin-lattice (*T*_1_) and increasing the speed of sound and ultrasound attenuation coefficient),^38,39^ glucose sensors, etc.) were key factors choosing copper oxide NSs among other MONSs. Moreover, the positive cytotoxic effect of copper oxide NSs on cancer cell lines can possibly be enhanced by functionalizing their surface with compounds possessing antitumor activity. Derivatives of α-aminophosphinic acid possess antitumor properties.^40^ Pyridine-containing phosphinic acids, which are used in medicine as new therapeutics for various diseases such as inflammation,^41^ asthma,^42^ diabetes,^43^ malaria,^44^ heart failure,^45^ human immunodeficiency virus (HIV),^46^ and cancer^47^ are very stable and effective bifunctional metal complexes with hepatotoxic activity (especially Cu(II) ions).^48^ They also show inhibitory activity toward bovine pancreatic chymotrypsin (12–25% inhibition).^49^ For these reasons, they represent a very interesting target for modern organic synthesis, and further evaluation of their physicochemical properties could provide valuable information on their potential applications.^50,51^ Also, the study of adsorption of the compounds, especially in the condensed phase where dissolved species accumulate at the interface between NSs and solution, is important for determining the adsorbate geometry with respect to the metal surface and the changes due to positional isomerism. Positional isomerism is known to affect the activity of compounds.^52^ For example, an analysis of the substitution pattern in Food and Drug Administration (FDA)-approved drugs (95 drugs) showed that the pyridine ring was mostly 4-substituted (*para*), in 11 drugs in the 2- and 6-position (*ortho*), and 1 drug had a monosubstitution in the 2-position. In addition, 3 drugs had monosubstitution in the 3-position and 10 drugs had disubstitution in the 3- and 5-position (*meta*).^53^

Accurate characterization of adsorbate behavior is critical because differences in the intensity of surface-enhanced Raman scattering (SERS) bands can be misinterpreted. From a medical perspective, changes in the intensity of the SERS signals are interpreted quantitatively (in terms of the adsorbate concentration) without taking into account the fact that the intensity of SERS bands depends on the orientation of the adsorbate on the metal surface. Such a misinterpretation may lead to diminishing the biological/medical significance of surface-functionalized NSs.

**Figure 1:**
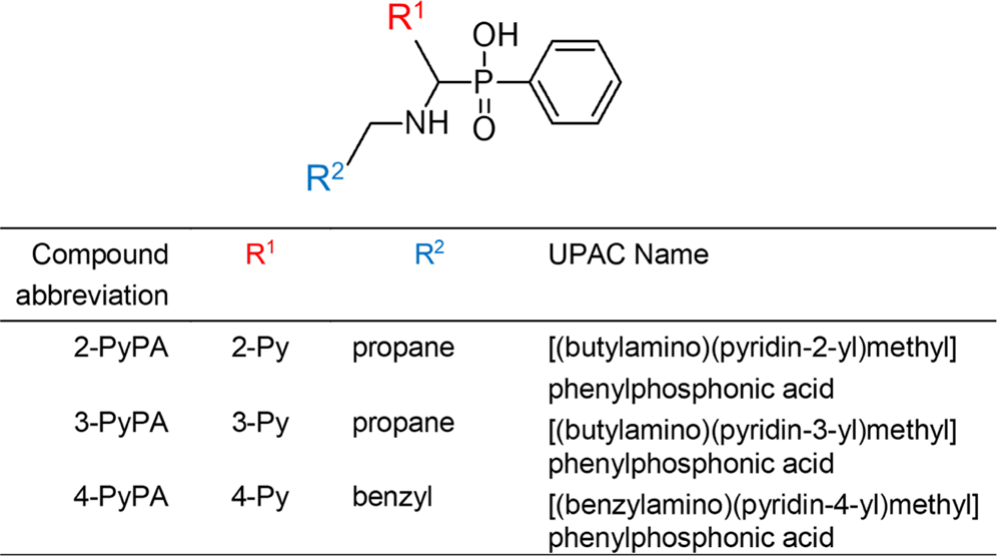
Structure of the investigated α-aminophosphinic acid derivatives of pyridine.

For the abovementioned medical reasons, methods are sought to prepare pure, physiologically stable, and reagent-free (to exclude any environmental interference) copper oxide NSs with a specific and controlled morphology. The present work fits seamlessly into this search and describes a method for the preparation of copper oxide NSs that meets the abovementioned criteria and is based on the anodic electrochemical dissolution of copper. The copper oxide NSs were characterized spectroscopically, and then three isomers of α-aminophenylphosphinic acids of pyridine, including [(butylamino)(pyridin-2-yl)methyl]phenylphosphinic acid (2-PyPA), [(butylamino)(pyridin-3-yl)methyl]phenyl-phosphinic acid (3-PyPA), and [(butylamino)(pyridin-4-yl)methyl]phenylphosphinic acid (4-PyPA) (see Figure 1 for the structures of these compounds), which have potential activity in cancer therapy, were synthesized and adsorbed onto their surfaces.

The resulting systems were characterized using the surface- and tip-enhanced Raman scattering techniques (SERS/TERS). Compared with other metal oxides, there are a limited number of reports on the use of unsupported copper oxide NSs as active substrates of SERS and for immobilization of small molecules or molecules with high symmetry.^54−58^ This is because aqueous copper oxide colloids are not as stable. This work shows for the first time how factors such as the oxidation state of the metal (Cu(I) vs Cu(II)), the contact interface (solid/water vs solid/air), incubation time, and positional isomerism [(butylamino)(pyridine)methyl]phenylphosphine (PyPA) affect adsorption. This work also shows that copper oxide NSs are not only effective substrates for SERS but can also act as potential drug carriers and sensitive (bio)sensors.

The studies at the solid/aqueous interface were carried out under physiological conditions, i.e., in the water environment. On the other hand, the studies at the solid/air interface were performed with a view to the practical application since not all measurements can be performed immediately (in the context of a large number of measurements or laboratory working hours) and/or nondrying of the sample is not always guaranteed (e.g., as a result of water evaporation due to heating of the sample (e.g., with laser radiation) or a long measurement period).

The shape and size of copper oxide NSs can be investigated by scanning/transmission electron microscopy (SEM/TEM), while X-ray diffraction (XRD) analysis provides information on the size and size distribution of crystalline domains. SERS allows the characterization of the adsorbate.^59−63^ The only question is whether the SERS signal is from the adsorbate localized at the surface of the NSs or at “hot spots”. Atomic force microscopy (AFM) combined with Raman spectrometry (TERS) answers this question. Thanks to nanometer resolution, TERS allows scanning the surface of the NSs and obtaining information about the geometry of the single molecule of the adsorbate.^64^

## Results and Discussion

### Characterization of Cu_2_ONPs

**Figure 2:**
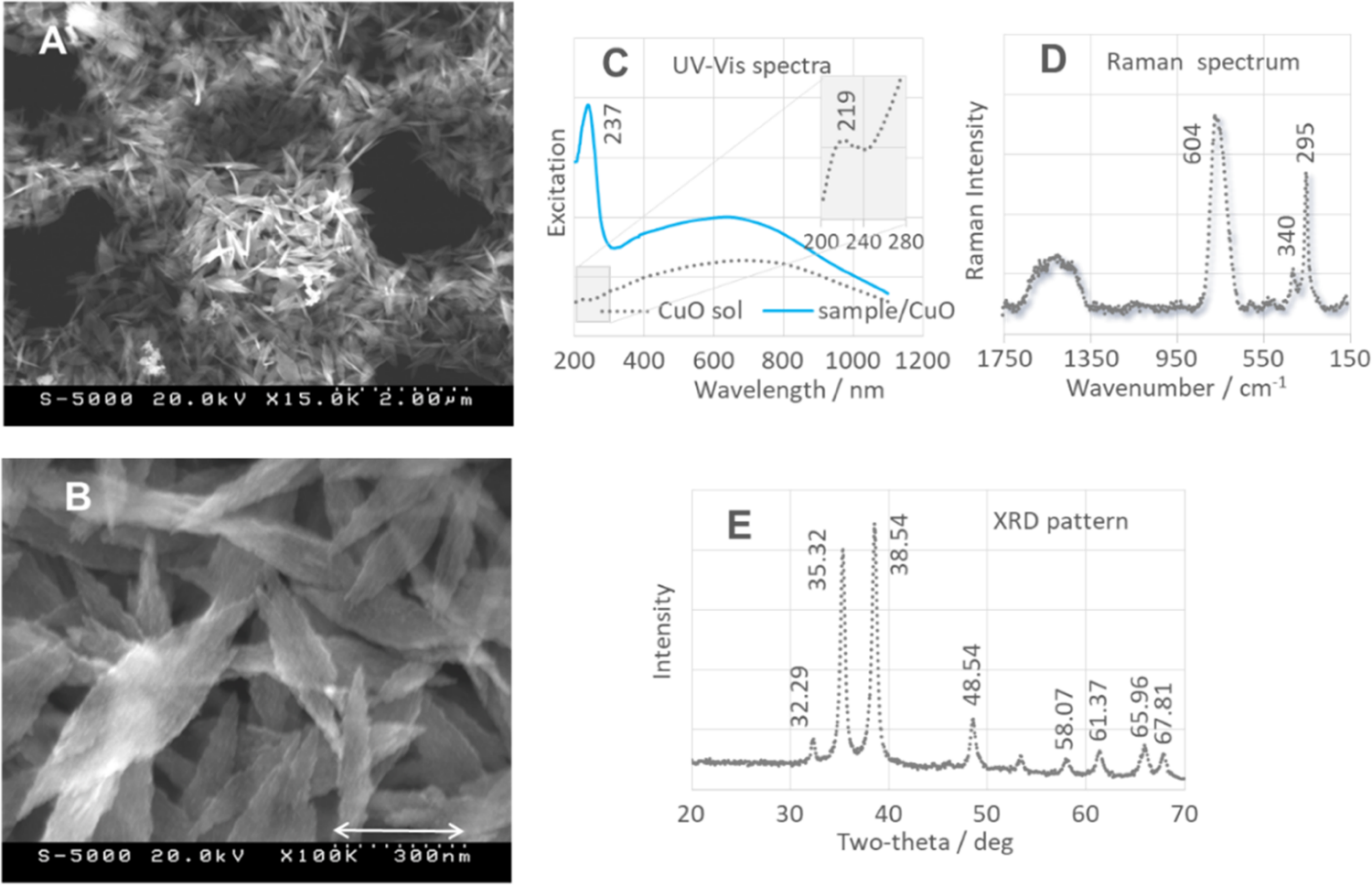
(A, B) SEM images of Cu_2_ONPs (measurement conditions: (A)—20.0 kV, 3.0k×, scale 10.0 μm and (B)—20.0 kV, 20.0k×, scale 1.5 μm), (C) excitation spectra (UV–vis) of Cu_2_ONPs (gray trace) and Cu_2_ONPs/sample (green trace) used in this work, (D) Raman spectrum of Cu_2_ONPs, and (E) XRD pattern of Cu_2_ONPs.

The SEM images (Figure 2A,B) show that the Cu_2_ONPs are spherical and have a size of 1.5 μm to 600 nm. This size of the Cu_2_ONPs is confirmed by the UV–vis spectrum, which shows two weak plasma resonances at 590 and 330 nm (Figure 2C, gray trace). The absorption at 590 nm is due to the band-gap transition of the CuO layer at the surface of the Cu_2_O nanocrystals.^65^ The absorption at 330 nm is due to the band-to-band transition in the nanocrystalline Cu_2_O (O^2–^:Cu^1+^ charge transfer (CT) band (O 2p → Cu 3d)).^66^

Cu_2_ONPs show characteristic Raman bands at 630 cm^–1^ (T_1u_), 486, 420, 219 (strongest E_u_), 182, and 148 (T_1u_ symmetry) cm^–1^ (Figure 2D) consistent with data from the literature.^66,67^ The 148 and 219 cm^–1^ bands are assigned to rotations of the Cu tetrahedron around its center. The 630 cm^–1^ spectral feature is due to out-of-plane vibrations of the Cu and O sublattice and is activated by defects (similar to the 148 cm^–1^ band).

The XRD spectrum of Cu_2_ONPs (Figure 2E) shows diffraction peaks at 2θ values ([*hkl*], crystallographic plane): 77.61 [(311)], 73.69 [(200)], 61.54 [(211)], 42.44 [(200)], 36.52 [(111)], and 29.60 [(110)] (*Pn*3*m*; JCPDS No. 78-2076), corresponding to a crystallographically pure, standard cubic cuprite structure.^68^

### Characterization of CuONSs

**Figure 3:**
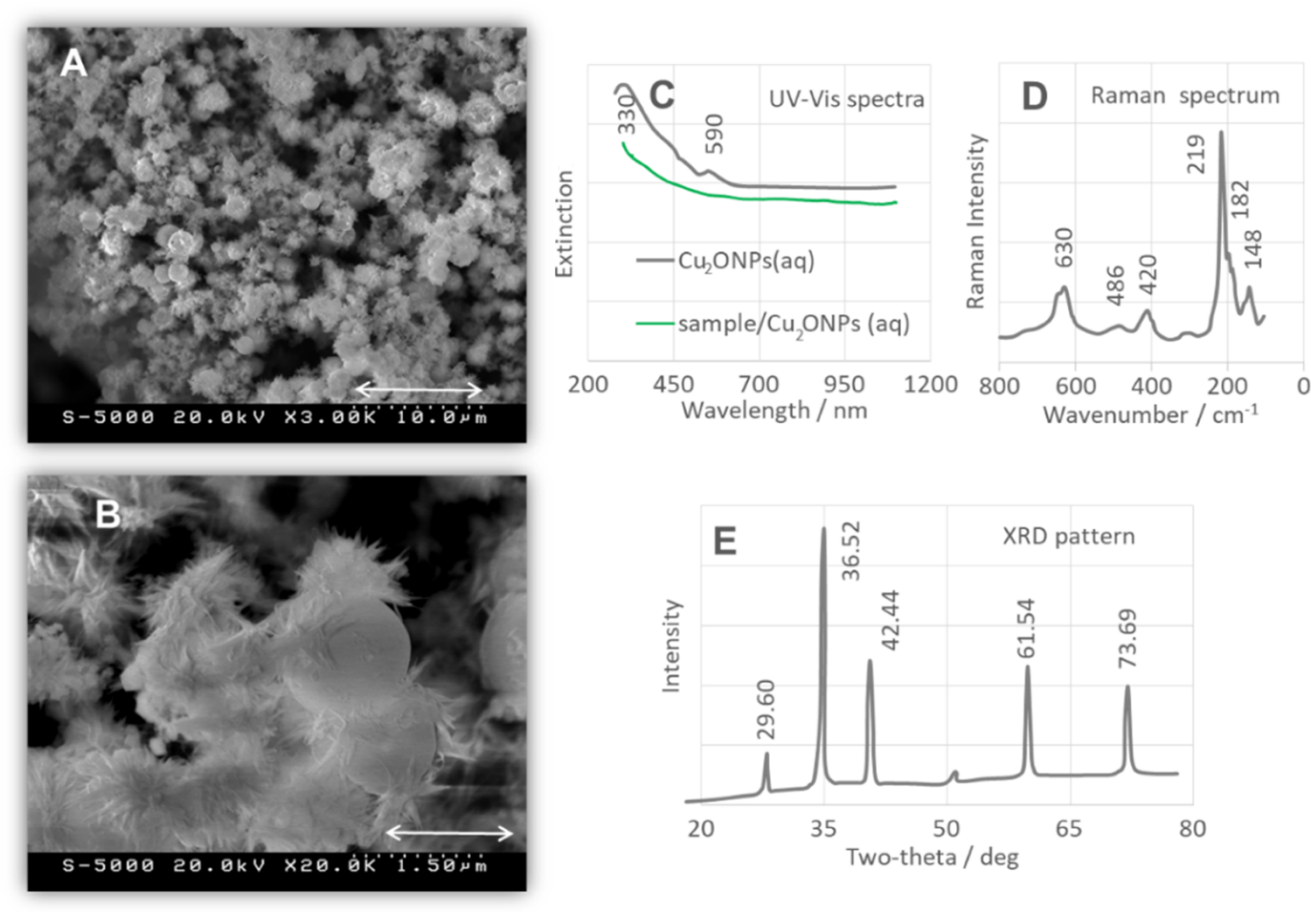
(A, B) SEM images of CuONSs (measurement conditions: (A)—20.0 kV, 3.0k×, scale 10.0 μm and (B)—20.0 kV, 20.0k×, scale 1.5 μm), (C) excitation spectra (UV–vis) of CuONSs (gray trace) and CuONSs/sample (green trace) used in this work, (D) Raman spectrum of CuONSs, and (E) XRD pattern of CuONSs.

The SEM images (Figure 3A,B) show that CuONSs have a leaf-like structure with average dimensions of 400–750 nm in length and 80–180 nm in width. The leaf-like structures consist of small self-aligned spherical particles (Figure 3B) and are confirmed by directional growth studies of CuO nanocrystals along the axis.^7,69^ CuONSs form a skeleton resembling a honeycomb structure and consist a network of pores with a submicrometer diameter (2–3 μm) and thickness (1–1.5 μm thick) (Figure 3A).

In the UV–vis spectrum of the bare CuONSs (Figure 3C, a gray dashed line), a weak band is observed at 219 nm, which is attributed to direct electron transfer.^70−72^ The spectrum of the sample/CuONSs (Figure 3C, a blue solid line), on the other hand, shows a broad absorption at 237 nm that is likely attributable to π–π* electronic transfer of the aromatic C=C groups of the molecule and/or CuONPSs···adsorbate electrostatic interaction.^73^

The Raman spectrum of CuONSs (Figure 3D) shows three active Raman modes at 604 cm^–1^ (B_g_), 340 (B_g_), and 295 (A_g_).^75^ These bands indicate the pure monoclinic CuO structure.^7,74^

The XRD spectrum of CuONSs (Figure 3E) shows diffraction peaks at 2θ values: 67.81, 65.96, 65.96, 61.37, 58.07, 53.40, 48,54, 38.54, 35.32, and 32.29°, which are assigned to the [220], [311̅], [113̅], [202, 020], [202̅], [111]/[200], [1̅11]/[002], and [110] planes of the pure CuO nanophase with a monoclinic structure (JCPDS No. 48-1548).^76^ The presence of highly crystalline CuONSs is confirmed by the significant intensity of the diffraction peaks.

### Adsorption Monitored by SERS

**Figure 4:**
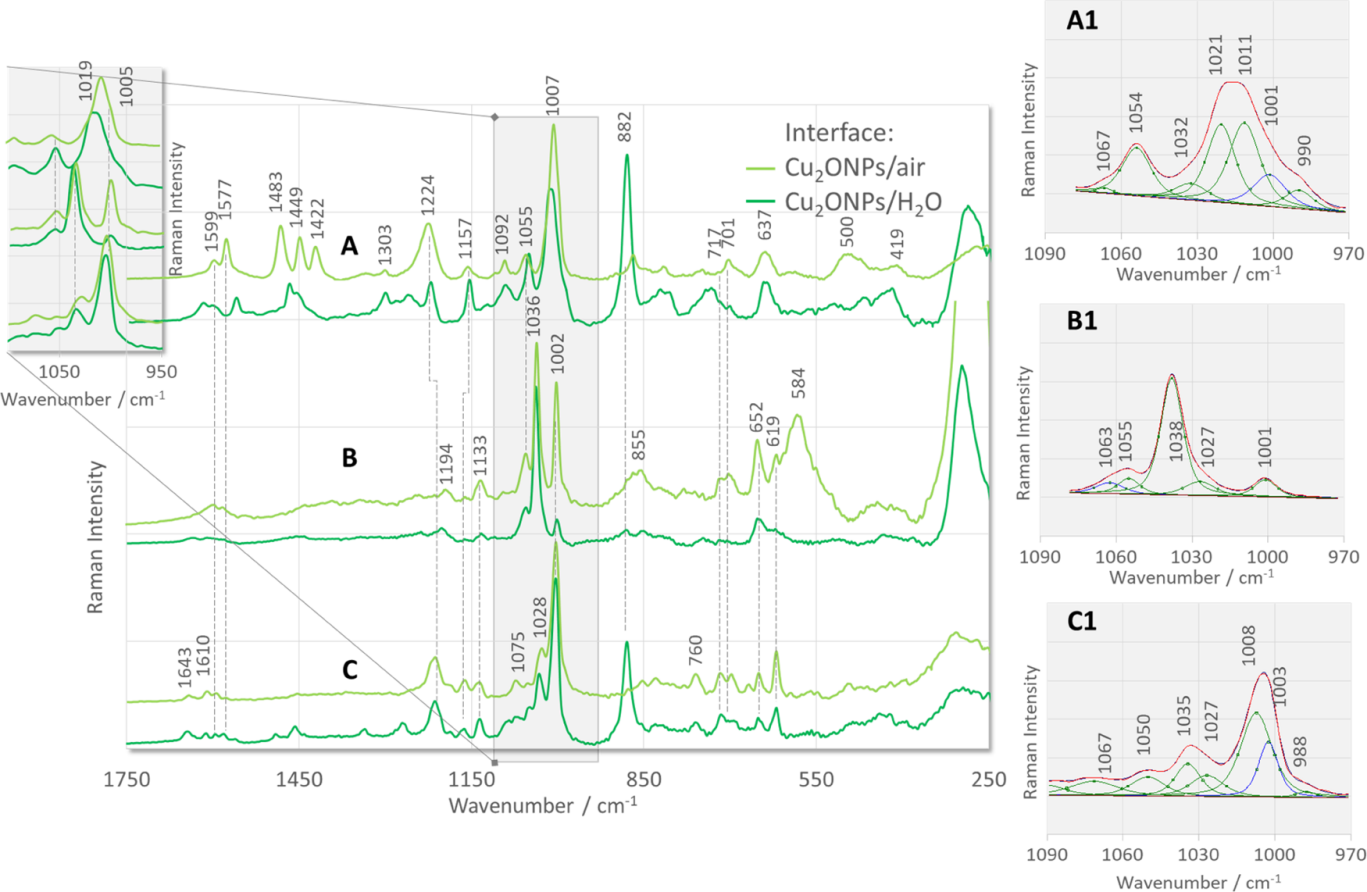
SERS spectra of the investigated 2- (A), 3- (B), and 4-isomers (C) of the pyridine α-aminophosphinic acid derivatives adsorbed on the Cu_2_ONPs/water (green (bottom) lines) and Cu_2_ONPs/air (aquamarine (upper) lines) interfaces (insets (A1)–(C1): fitting results of 2-, 3-, and 4-isomer spectra, respectively, in the spectral region of 970–1090 cm^–1^).

**Figure 5:**
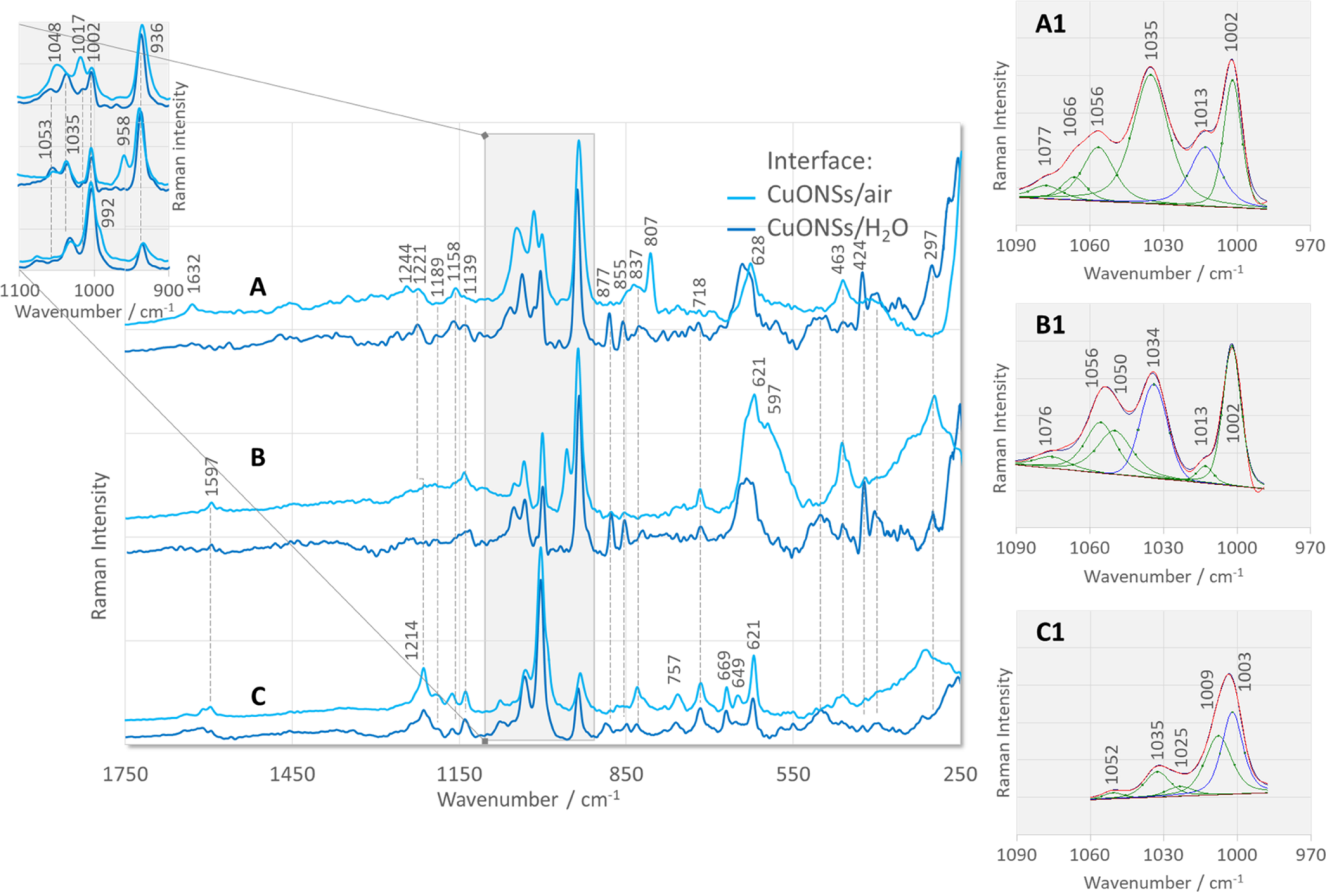
SERS spectra of the investigated 2- (A), 3- (B), and 4-isomers (C) of the pyridine α-aminophosphinic acid derivatives adsorbed on the CuONSs/water (navy (bottom) lines) and CuONSs/air (blue (upper) lines) interfaces (insets (A1)–(C1): fitting results of 2-, 3-, and 4-isomer spectra, respectively, in the spectral region of 970–1090 cm^–1^).

Figure 4 shows the SERS spectra of the three isomers (2-, 3-, and 4-) of the derivative of pyridine α-aminophosphinic acid adsorbed at the Cu_2_ONPs/H_2_O (Figure 4, green lines) and Cu_2_ONPs/air (Figure 4, aquamarine lines) interfaces. For comparison, the SERS spectra of these compounds adsorbed at the CuONSPs/water (navy lines) and CuONSs/air (blue lines) interfaces are shown in Figure 5. The proposed assignment of the bands to the normal modes describing the different vibrational motions in the molecule is summarized in Table 1. The given band assignment is not discussed in detail because an analysis of the potential energy distribution (PED) based on density functional theory (DFT/B3LYP6-311G(df,p)) has already been performed.^77^ The band analysis presented also includes literature data on phenyl- and pyridine-substituted molecules.^78−81^

**Table 1:** Wavenumber of Selected Bands of the α-Aminophosphinic Acid Derivatives of Pyridine Adsorbed at the CuONSs/Water Interface^a^

	wavenumbers (cm^–1^)		
bands assignment^a^	2-PyPA	3-PyPA	4-PyPA
ν_1Py_ + ν_6bPy_	1620	1636	1643
ν_8aPy_	1603	1608	1610
ν_8aPh_		1599	1597
ν_8bPh_		1582	1580
	1563		1562
Ph	1471	1478	
Py	1458	1454	1457
ν_19bPh_, δ(CH_2_/CH_3_)	1453	1442	
ν(P=O), δ(C–C–H)	1264	1239	1268
ν_3Py_	1225	1201	1215
ν_9aPy_, ν_7a_[C–C_Ph_], ν_9aPh_		1194	1188
ν(P=O), δ(CH)	1157	1160	1164
ν(P–OH)	1138	1133	1135
ν_18aPy_	1067^d^	1063^d^	1067^d^
ν_18bPy_	1054^d^	1055^d^	1050^d^
ν_12Py_	1032^d^	1038^d^	1035^d^
ν_18aPh_	1021^d^	1027^d^	1027^d^
ν_1Py_	1011^d^		1008^d^
ν_12Ph_	1001^d^	1001^d^	1003^d^
	990^d^		988^d^
ρ_b_(POH)	882	882	882
ν(P–O)	854	855	855
	825		830
δ(Ph)	761		760
ν_4Py_, ν(P–C_Ph_), ρ_as_(Ph), ν(P–C), δ(PCC_Ph_)	738		716
ν_6bPy_, ρ_as_(Py) + δ(C_pyr_CP)	645	652	651
ν_6bPh_	623	619	621
Cu···OH	459		463
Cu···N	289		291

a Py, the pyridine ring; Ph, the phenyl ring; ν, stretching; δ, deformation; ρ_t_, twisting; oop, out of plane; and as, antisymmetric vibrations; d, fitted bands.

The p*K*_a_ values of the phosphinic acid group and the pyridine are below 6 and above 10, respectively. Therefore, the neutral form of these groups is expected to be present in the Cu_2_ONPs sol at pH = 7, which is confirmed by the bands at 882 and 1138 cm^–1^ (see Table 1 for band assignment) in the spectra of the studied isomers (Figure 4, green lines).^82,83^ The first of these bands is weakly enhanced in the Raman spectra of all of the isomers studied, while the second band has intermediate relative intensity (the intensity of the band is given with respect to the band with the highest intensity). The relative intensities of these bands differ in the SERS spectra. Briefly, in the 2-PyPA SERS spectrum at the Cu_2_ONPs/H_2_O interface (Figure 4A, green line), the 882 cm^–1^ band is the most intense band in the spectrum. In the 3-PyPA spectrum (Figure 4B, green line), this band is slightly enhanced, while in the 4-PyPA spectrum (Figure 4C, green line), it has about 60% of the intensity of the band in 2-PyPA. The second from the mentioned bands is weak in the spectra of all isomers (slightly enhanced only in 2-PyPA) and weaker than in the Raman spectra.

For strong band enhancement to occur at 882 cm^–1^ (in the case of 2-PyPA at Cu_2_ONPs/H_2_O), the lone pair of electrons on the oxygen atom (sp^3^ hybridization) must be in direct contact with the surface of the nanoparticle, and therefore the sp^3^ orbital occupied by the lone pair of electrons should be perpendicular to the substrate surface. This means that both the O–H bond and the O–P bond deviate from the normal to the surface of the substrate by an angle of 71°, i.e., the bonds are more or less parallel to the surface of the substrate (angle of 19°). Therefore, a very weak enhancement of the ν(P–O) mode can be expected for this isomer, which is observed. The decrease in the intensity of the 882 cm^–1^ band and a weak intensity of the 1135 cm^–1^ band for 4-PyPA compared to 2-PyPA could indicate a deviation of the sp^3^ oxygen orbital with a lone pair of electrons with respect to the normal surface so that the angle between the O–P bond and the normal surface of the substrate decreases (i.e., the hydrogen atom approaches the surface of the substrate). In the case of the 3-PyPA isomer, the low intensity of the two bands discussed can be explained by the distance effect. That is, the phosphinic acid group is far from the surface of the substrate.

**Figure 6:**
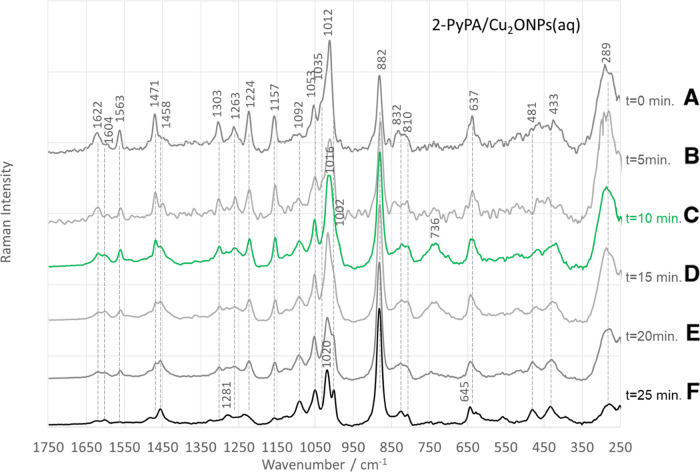
Time-dependent SERS spectra of the 2-PyPA isomer of the pyridine α-aminophosphinic acid derivative adsorbed on the Cu_2_ONPs/H_2_O interface (measured immediately after adsorption (*t* = 0 min) (A), *t* = 5 min (B), *t* = 10 min (C), *t* = 15 min (D), *t* = 20 min (E), and *t* = 25 min (F)).

In the case of 2-PyPA at the Cu_2_ONPs/H_2_O interface, the intensity of the 882 cm^–1^ band depends on the incubation time of the isomer after mixing with the colloid (Figure 6), implying that the molecule on this interface reorients with time so that the O–H bond, which was originally practically parallel to the substrate surface, lifts to some extent relative to it. This can be seen in the following spectral changes. In the spectrum measured immediately after mixing (*t* = 0 s), the 882 cm^–1^ SERS signal is the second strongest band in the spectrum (after the 1012 cm^–1^ band) and has about 70% of the intensity of the 1012 cm^–1^ spectral feature. In the spectrum measured after 5 and 10 min of incubation, the 882 cm^–1^ band increases by about 30% each time. Further extension of the incubation time (≥10 min) does not lead to any further changes in the enhancement of this band. For this isomer adsorbed on the surface of CuONSs (Figure 5A) and for the other isomers (3- and 4-) deposited on Cu_2_ONPs (Figure 4B,C) and CuONSs (Figure 5B,C), the intensity of the 882 cm^–1^ band does not change with time. The orientation changes observed only for 2-PyPA are puzzling. Only for this isomer, the distance between the pyridine nitrogen atom and the phosphine oxygen atom, i.e., two atoms carrying a lone pair of electrons and therefore having a high affinity for a substrate surface, is the shortest, so that an intermolecular N_Py_···HO_phosphinic_ hydrogen bond can be formed. The breaking of this bond due to the interaction with the Cu_2_ONPs surface can be assumed as a probable explanation for the observed changes.

When the Cu_2_ONPs/H_2_O interface (Figure 4, green lines) is replaced by Cu_2_ONPs/air (Figure 4, aquamarine lines), the intensity of the 1135 cm^–1^ band practically does not change and remains weak or very weak for all isomers studied, while the 882 cm^–1^ band is absent. This could be an indication of the deprotonation of the phosphinic acid group.

When the oxidation state of copper changes from Cu(I) to Cu(II), the type of interface (CuONSs/H_2_O (Figure 5, navy lines) vs CuONSs/air (Figure 5, blue lines)) at which the molecules are adsorbed has a smaller effect on the behavior of the 877 cm^–1^ band; this spectral feature shows low intensity for all of the isomers at the CuONSs/H_2_O interface (*I*_877 2-PyPA_ ≅ *I*_877 3-PyPA_ > *I*_877 4-PyPA_) and disappears at CuONSs/air. Thus, the isomerism of the substituent has practically no effect on the relative intensity of this band for molecules adsorbed on the surface of CuONSPs.

**Figure 7:**
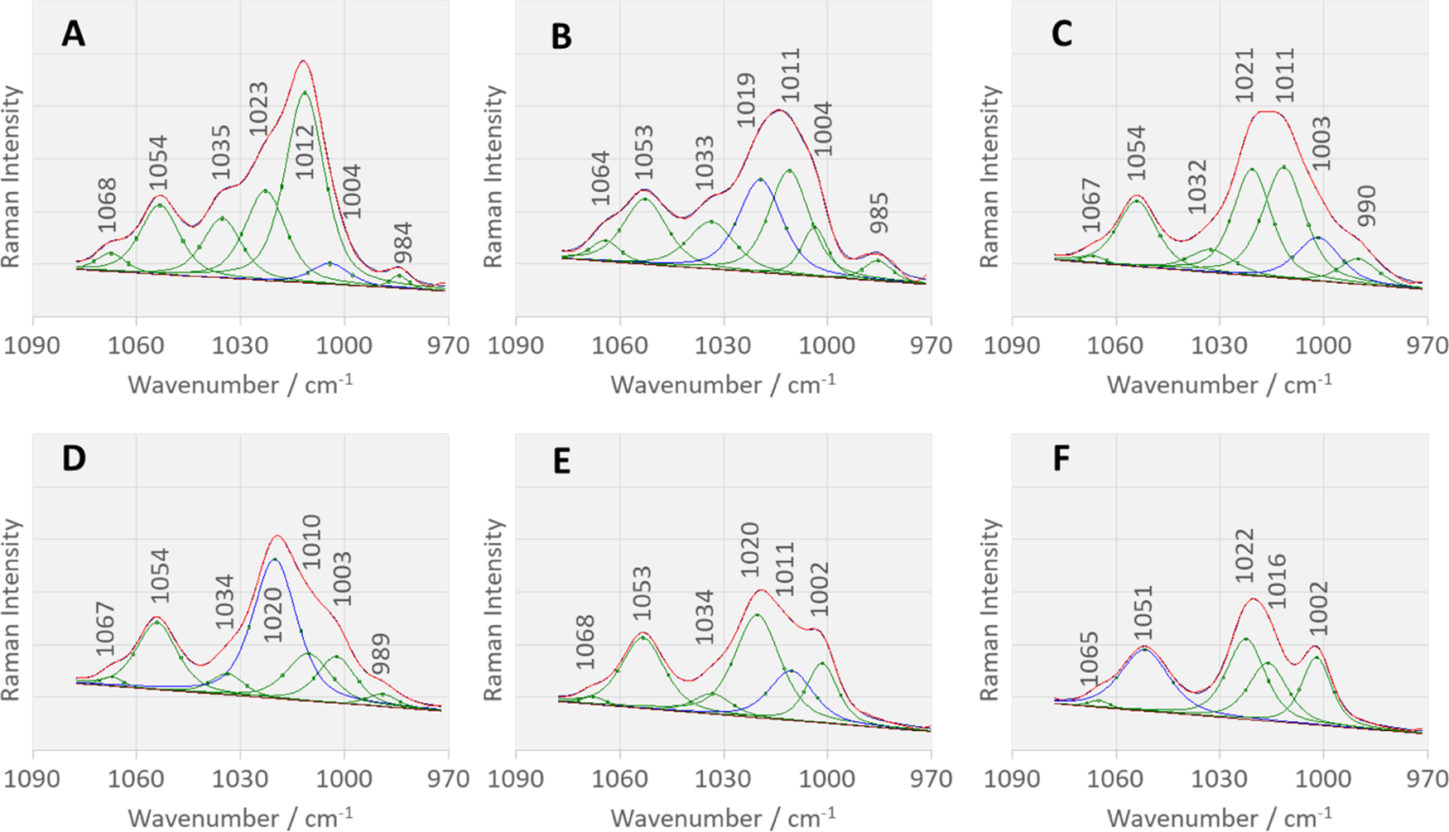
Fitting results of the time-dependent SERS spectra of the 2-PyPA isomer of the pyridine α-aminophosphinic acid derivative adsorbed on the Cu_2_ONPs/H_2_O interface (measured immediately after adsorption (*t* = 0 min) (A), *t* = 5 min (B), *t* = 10 min (C), *t* = 15 min (D), *t* = 20 min (E), and *t* = 25 min (F)).

Time-dependent changes can also be seen in the other bands, of which those in the spectral range from 970 to 1080 cm^–1^ are of importance as they allow the determination of the change in the molecule–surface interaction. Within the abovementioned wavenumber range, seven bands can be identified (see Figure 7), which have been assigned to the vibrations of phenyl (ν_12Ph_ and ν_18aPh_) and pyridine rings (ν_1Py_ (trigonal ring breathing involving C_3_NC_5_ atoms), ν_12Py_ (trigonal ring breathing involving C_2_C_4_C_6_ atoms) and ν_18bPy_).^84^ Since we know that in the case of monosubstituted pyridine, the ν_12_ mode (e.g., the 3-isomer) or the ν_1_ mode (e.g., the 2- and 4-isomers) becomes more dominant in the Raman spectra, and the ν_1_ mode is not enhanced for the 3-isomer;^85^ as can be seen in the SERS spectrum in Figure 4B (the fitting results for the isomer 3-), the following assignments of the abovementioned modes to bands can be made: 1002, 1020, 1010, 1034, and 1053 cm^–1^.

In the 2-PyPA SERS spectrum at Cu_2_ONPs/H_2_O for *t* = 0 min. (Figure 6A), the ν_12_ mode of the phenyl ring (ν_12Ph_) is weak, which may indicate that the phenyl ring is parallel to the surface of the substrate compared to its high intensity in the Raman spectrum. This is confirmed by both the shift in its wavenumber (Δν = +5 cm^–1^) and the broadening of its bandwidth (Δ_FWHM_ = +6 cm^–1^) compared to those in the Raman spectrum. The intensity of this band increases with increasing incubation time each time, up to an intensity corresponding to three times its initial intensity (*t* = 25 min, Figure 6F). Thus, it can be assumed that the phenyl ring increases relative to the Cu_2_ONPs/H_2_O interface.

The increase in ν_12Ph_ intensity with increasing incubation time is accompanied by a change in the intensity of the SERS signals at 1012 and 1035 cm^–1^. The 1012 cm^–1^ band is the strongest in the SERS spectrum at *t* = 0 min (Figure 6A). In the SERS spectra at *t* = 5 and 10 min (Figure 6B,C), it has comparable intensity but loses 40% of intensity compared to the SERS spectrum at *t* = 0 min. In the spectra for a longer incubation time, i.e., *t* = 15 and 20 min (Figure 6D,E), it again loses about 50% of intensity. For a time *t* = 25 min (Figure 6F), it increases by about 15%. Jagodzinski and colleagues divided the spectra of pyridine adsorbed on various metal surfaces into two groups on the basis of the behavior of the bands at 1036, 1008, and 654 cm^–1^.^84^ In the spectra of the first group (end-on absorption orientation of Py on Ag), the bands are enhanced at 650 and 1036 cm^–1^, with the 1036 cm^–1^ band having at least 50% of the intensity of the band at 1008 cm^–1^. In the spectra of the second group (edge-on adsorption geometry of Py on Cu), the band at 1036 cm^–1^ is weak (it has at most 25% of the intensity of the band at 1008 cm^–1^), and the band at 650 cm^–1^ is absent. Moreover, in the spectra of the latter group, the SERS signals at 1213, 1597, and 1640 cm^–1^ are stronger than in the spectra of the “end-on” group. The authors found that the 654 cm^–1^ SERS signal (B_1_ point symmetry group) disappears at the copper surface and the 633 cm^–1^ spectral feature (A_1_) appears. These authors also found that pyridine achieves equilibrium between “end-on” and “edge-on” (α-pyridyl) orientations on other metal surfaces, resulting in intermediate spectra between the spectra on Ag and Cu surfaces. Based on the abovementioned studies, it can be assumed that both forms of pyridine are present at the Cu_2_ONPs/H_2_O interface and that the equilibrium between these two forms in the first minutes after adsorption is shifted toward the “edge-on” form (at *t* = 0 min, intensity ratio *I*_1012_/*I*_1023_ = 3.5 and bands at 637, 1224, 1563, and 1622 cm^–1^ are stronger than at *t* = 25 min) and shifts toward the “end-on” form with increasing incubation time (at *t* = 25 min, intensity ratio *I*_1012_/*I*_1023_ = 2.1 and the band at 645 cm^–1^ is stronger than at *t* = 0 min). The intense SERS signal at 289 cm^–1^ min, which is due to Cu–N vibrations, can confirm this statement. The enhancement of this mode is strong at *t* = 0 min and decreases when the 2-PyPA molecule rotates at the edge and the pyridine turns into α-pyridyl. Therefore, the differences between the SERS spectra of 2-PyPA at different incubation times are due to the degree of conversion of pyridine to α-pyridyl species.^86^

The change of the interface type from Cu_2_ONPs/H_2_O to Cu_2_ONPs/air leads to some changes in the spectral profile of the pyridine bands in the SERS spectrum of 2-PyPA (Figure 4A). The most important changes include the enhancement of the medium-intensity bands at 1577, 1483, 1449, and 1422 cm^–1^, the attenuation of the spectral features at 1157 and 1055 cm^–1^, the shift of the wavenumber of ν_8bPy_ and ν_1Py_, the disappearance of the 1622 cm^–1^ SERS signal, and the significant broadening of the band at 1224 cm^–1^. These changes can be explained on the basis of the work of Uvdal et al.^86^ These authors have shown that in the SERS spectrum of pyridine, when the ring is arranged vertically and tilted on edge relative to the substrate surface, the bands due to A_1_ and B_1_ symmetry are mainly enhanced, whereas when the ring is tilted frontally, the A_1_ and B_2_ symmetry modes are mainly enhanced. In the present case, the 1157 and 1055 cm^–1^ bands are due to the B_2_ symmetry modes, while 1577, 1483, 1449, 1422, and 1212 cm^–1^ SERS signals are due to the B_1_ symmetry modes. Thus, the attenuation of the B_2_ modes and the enhancement of the B_1_ modes indicate a perpendicular arrangement of the pyridine ring, which is in contact with the substrate surface through its C–N bond.

In the spectrum of 3-PyPA on Cu_2_ONPs (Figure 4B), a shift in the wavenumber of the bands in the blue direction is observed between the Raman and SERS spectra. The ν_12Py_ and ν_3Py_ modes are shifted from 1027 and 1201 cm^–1^ in the Raman spectrum to 1038 and 1224 cm^–1^ in the SERS spectrum. Similarly, in the 4-PyPA spectrum (Figure 4C), the ν_1Py_ mode is shifted from 1004 cm^–1^ in the Raman spectrum to 1008 cm^–1^ in the SERS spectrum. Suh et al. have shown that for isomers of pyridine carboxylic acid, the wavenumber shift of the pyridine modes due to adsorption on the surface is relatively small when the interaction with the metal surface through the nitrogen atom is weak.^87^ This can be explained by the fact that the surface geometry of the pyridine ring is slightly inclined to the surface. The large shift of 11 cm^–1^ observed for 3-PyPA at Cu_2_ONPs/H_2_O correlates with higher vibrational energy and is indicative of a strong coordination bond (pyridine ring perpendicular to the Cu_2_ONPs/H_2_O interface). On the other hand, the 4 cm^–1^ shift in the wavenumber for 4-PyPA indicates that the pyridine ring is tilted with respect to the substrate surface compared to fully planar geometry. In a planar geometry, where no coordination bonds are formed, shifts are less likely because the bond between the pyridine ring and the surface is also much weaker and mostly physical.

Comparison of the fitting results of the spectra of 3-PyPA and 4-PyPA at the Cu_2_ONPs/H_2_O interface (Figure 4B1,C1) with the corresponding Raman spectra shows that the phenyl ring adopts a nearly parallel orientation on this interface for 3-PyPA (the 1001 and 1027 cm^–1^ SERS signals are weak) and is tilted for 4-PyPA (the 1003 and 1027 cm^–1^ bands are medium strength). Interestingly, the 4-PyPA isomer contains two phenyl rings in its structure, but only the vibrations of one of these rings are observed. Considering the optimized structure of this molecule^77^ and the fact that the ρ_b_(POH) mode in the SERS spectrum of 4-PyPA is observed at the Cu_2_ONPs/H_2_O interface, it can be assumed that this ring is a phenyl ring in the phosphinic acid group.

In the case of the 3-PyPA isomer, the change in the contact interface (Cu_2_ONPs/H_2_O → Cu_2_ONPs/air) (Figure 4) mainly leads to a realignment of the phenyl ring from a practically parallel to a practically vertical position with respect to the surface, as evidenced by a significant increase in the band intensity at 1002 cm^–1^ (the second strongest band in the spectrum). In the case of 4-PyPA, only a slight attenuation of the intensity of some bands of the aromatic ring vibrations is observed. On this basis, it can be assumed that both the phenyl ring and the pyridine ring do not reorient. The change in the oxidation state of copper also affects the spectral profile so that the most intense bands in the spectra are at 936 and 1002 cm^–1^. The 936 cm^–1^ spectral feature is most pronounced in the SERS spectra of 2-PyPA and 3-PyPA. This band is accompanied by 463 and 267 cm^–1^ spectral features and increasing and broadening bands in the spectral region between 500 and 650 cm^–1^. These observations can be explained by the assumption that the CuONS modes (604 and 295 cm^–1^ (Figure 4)) overlap with the bands originating from the 2- and 3-PyPA modes and that protons are bound to the CuONS surface, leading to SERS signals at 936 [ν(Cu–OH)] and 490–460 cm^–1^.^88−90^

The fitting procedure in the spectral region of 980–1090 cm^–1^ of the SERS spectra of the three discussed isomers adsorbed on the surface of CuONSs (Figure 5A1–C1) proves that these isomers interact with this surface via the pyridine and phenyl rings. However, the arrangement of these rings relative to the surface of CuONSs is altered compared to Cu_2_ONPs. In the case of 2-PyPA at the CuONSs/H_2_O interface, the intensity of the 1001 cm^–1^ band is higher than the intensity of the 1013 cm^–1^ SERS signal, while at the CuONSs/air interface, these intensities are reversed (*I*_1013_ > *I*_1002_). In the SERS spectrum of 3-PyPA, the 1013 cm^–1^ band is weak (at CuONSs/H_2_O) or absent (at CuONSs/air) (Figure 5A1,B1). For 4-PyPA, the ν_1_ mode is hidden under the most pronounced 1003 cm^–1^ SERS signal and the wavenumber shifts upward when the molecule is immobilized at the CuONSs/H_2_O interface or downward when immobilized at the CuONSs/air interface. From the abovementioned observations and the changes in the wavenumber of the SERS bands relative to the Raman bands (Δν_12Ph_ = +3, +5, and +4 cm^–1^ for 2-, 3-, and 4-PyPA and Δν_1Py_ = 21 cm^–1^ for 2-PyPA, Δν_12Py_ = 8 cm^–1^ for 3-PyPA, and Δν_1Py_ = 5 cm^–1^ for 4-PyPA), it can be concluded that the phenyl ring is tilted with respect to the CuONS surface for 2-PyPA and 3-PyPA and practically vertical for 4-PyPA and that its arrangement does not change practically when the interface changes from CuONSs/H_2_O to CuONSs/air for the 3-PyPA and 4-PyPA isomers, while the phenyl ring is slightly more inclined for the 2-PyPA isomer. One could also speculate that the pyridine ring adopts an approximately vertical orientation with respect to the CuONSs/H_2_O interface for 2-PyPA and a tilted one for the other isomers. In the case of the 4-PyPA isomer, the pyridine ring is less deviated from the surface normal and interacts with the surface via the pyrrole nitrogen (649 cm^–1^, “end-on” orientation), while the broad band at 634 cm^–1^ for −PyPA might be related to the presence of pyridine in an equilibrium between “end-on” and “edge-on” orientation.

### TERS Studies

**Figure 8:**
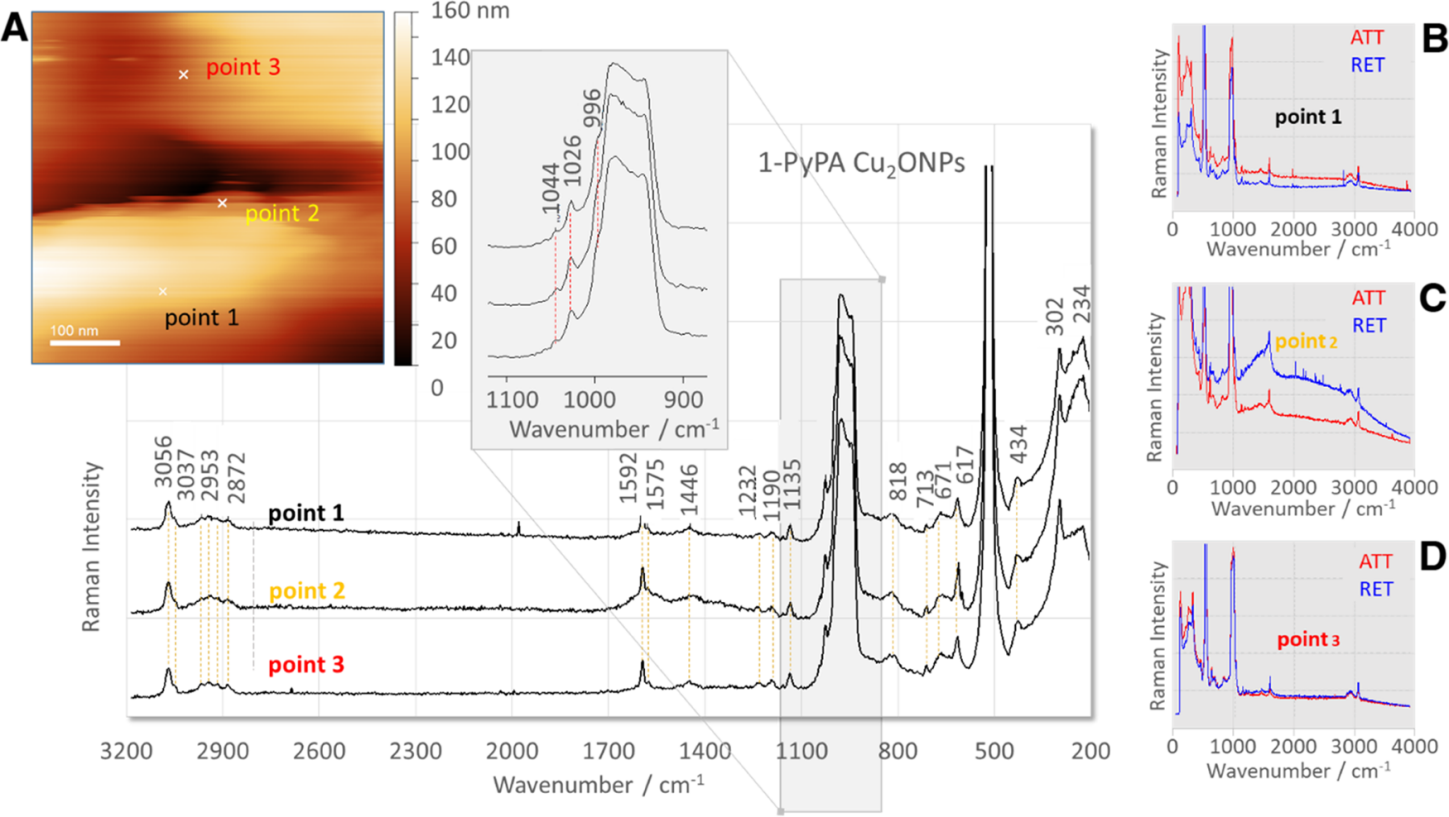
TERS spectra of the 2-PyPA isomer adsorbed on the Cu_2_ONP surface (insets: (A) AFM image of Cu_2_ONPs with marked measurement points and (B–D) spectra measured under the conditions of the approached (red traces) and retracted (blue traces) tip at measurement points 1, 2, and 3, respectively).

Figure 8A shows an AFM image of the Cu_2_ONP surface (scale bar 100 nm) with the three selected measuring points, where the TERS spectra of the 2-PyPA isomer were collected. Figure 8 shows the TERS spectra for the three abovementioned points obtained by subtracting the spectrum measured under the retracted tip conditions from the spectrum acquired under the approached tip conditions (insets B–D). These TERS spectra show the bands characteristic of the weakly scattering 2-PyPA molecule, which are hardly detectable using normal Raman spectroscopy. Comparison of the TERS spectra at all measuring points shows good reproducibility of the measurements. The intensities of the bands in the TERS spectra recorded at the different measurement points differ only slightly. This is not surprising given that the size of Cu_2_ONPs is very large compared to the size of the molecule in contact with the Cu_2_ONPs. Thus, the molecule “fills” the surface of the Cu_2_ONPs like a flat surface.

Comparison of the TERS spectra of 2-PyPA with the corresponding SERS spectrum at the Cu_2_ONPs/air interface (Figure 4A, aquamarine line) shows the same series of bands (1597, 1575, 1446, 1232, 1190, 1135, 1044, 1026, 996, 818, 713, 671, 617, 424, and 302 cm^–1^). However, the TERS signals are significantly enhanced and narrower compared to the corresponding SERS signals. Moreover, the TERS spectra are free from ambient interference and support the predicted mode of 2-PyPA adsorption mainly through the vertical α-pyridyl ring, and the phenyl ring and the deprotonated phosphine group are located near the surface of the substrate, which were submerged based on the SERS spectrum.

### SERS Enhancement

The enhancement factor (EF) evaluates the effectiveness of the SERS substrate. The enhancement factor is often expressed as EF = (*I*_SERS_/*c*_SERS_)/(*I*_RS_/*c*_RS_), where *I*_SERS_ and *I*_RS_ are the band intensities in the SERS and Raman spectra, respectively, and *c*_SERS_ and *c*_RS_ are the concentrations of the analyte used for SERS and Raman measurements, respectively.^91^ For equal analyte concentrations, EF equas *I*_SERS_/*I*_RS_. The EF determined is up to 10^6^ orders of magnitude for Ag and Au@SiO_2_; 10^5^ orders of magnitude for Au; 10^4^ orders of magnitude for Cu and Ti; 10^3^ orders of magnitude for ZnO, CuO, Cu_2_O, TiO_2_, and γ-Fe_2_O_3_; and 10^2^ orders of magnitude for Zn and Fe.^92,93^

Using the SERS spectra, the mechanism of enhancement can be predicted for the analyte and the substrate. The mechanism of enhancement can be predicted from the SERS spectra. The SERS spectrum of the molecule physically adsorbed on the metal surface (electromagnetic (EM) mechanism) is similar to the Raman spectrum of the free molecule.^94,95^ The SERS spectrum of the molecule chemically adsorbed on the metal surface (charge transfer (CT) mechanism) changes drastically in the wavenumber and intensity compared to the corresponding Raman spectrum. This is because the adsorbate–molecule complex leads to drastic changes in the wavenumbers and intensities of the SERS bands of the adsorbate.^96^ Otero et al. have shown that the CT mechanism causes the enormous SERS intensity of the ν_8a_ vibration. For this reason, in aromatic ring-containing adsorbates (e.g., pyridazine, pyridine, benzene, and their derivatives), it can be used as a marker band of enhancement by the CT mechanism.^96−98^ Based on the abovementioned information and the fact that in the case of the SERS spectra of PyPA, no strong enhancement of the ν_8a_ mode is observed and the differences in wavenumber, intensity, and width of the SERS vs Raman bands of PyPA are small, it can be concluded that on the tested substrates, the EM mechanism is responsible for the signal enhancement.

## Conclusions

In this work, Cu_2_O nanoparticles (Cu_2_ONPs) (spherical with an average size of 1500–600 nm; crystallized in a cubic cuprite structure) and the leaf-like nanostructures (CuONSs) (average size of 80–180 nm in width and 400–750 nm in length; crystallized in a monoclinic structure) were synthesized by anodic electrochemical dissolution of copper in ethanol or an aqueous solution with a LiCl electrolyte. The morphology, size, and structure of the copper oxide NSs were verified by SEM, XRD, UV–vis, attenuated total reflection-Fourier transform infrared spectroscopy (AT-FTIR), and Raman spectroscopy (RS). The mode of adsorption of the 2-, 3-, and 4-isomers of α-aminophosphinic acid derivatives of pyridine immobilized at Cu_2_ONPs/air and Cu_2_ONPs/aqueous solution and CuONSs/air and CuONSs/aqueous solution interfaces was observed at pH 7 at an excitation wavelength of 785.0 nm.

The investigations performed in this study showed that the oxidation state of the metal (Cu(I) vs Cu(II)), the nature of the contact interface (solid/water vs solid/air), and the positional isomerism of the adsorbate affect the geometry of the molecule on the copper oxide NS surface. In the case of the 2-isomer, the incubation time also affects the adsorption of this molecule at the Cu_2_ONPs/H_2_O interfaces, which can be explained by the breaking of the intermolecular N_Py_···HO_phosphinic_ hydrogen bond under the influence of adsorption. For molecules adsorbed on the Cu_2_ONPs surface, many more changes are observed under the influence of the interface type (from Cu_2_ONPs/H_2_O to Cu_2_ONPs/air) than on the CuONS surface. That is, for 2-PyPA and 3-PyPA, the change of the contact interface causes not only changes in the orientation of the aromatic rings but also deprotonation of the phosphinic acid group.

The TERS spectra of 2-PyPA immobilized on Cu_2_ONPs recorded from different points on the surface show a high degree of similarity. This is because the molecule “fills” the Cu_2_ONP surface like a flat surface. In addition, the TERS measurements avoided interference from the surrounding environment, which obscures the SERS spectrum, thanks to the nanometer spatial resolution.

These studies have demonstrated the usefulness of Cu_2_ONPs and CuONSs as effective sensors for the studied compounds with anticancer activity. Thus, the process of catalytic destruction of tumor cells could be enhanced by the introduction of copper oxide NSs with active compounds immobilized on their surface.

## Experimental Section

### Synthesis of Phenylphosphinic Acids

α-Aminophenylphosphinic acids of pyridine (Figure 1) were synthesized by the addition of silylated phosphoric acid esters to suitable pyridinimines.^99^ The detailed synthetic procedure and NMR characterization of these compounds have been published previously.^100^ All of the pyridine α-aminophenylphosphinic acids used in this study are present as racemic mixtures. The purity and chemical structures of the studied compounds were confirmed by ^1^H, ^13^C, and ^31^P NMR spectra recorded on a JEOL 400yh instrument (400 MHz for ^1^H NMR, 162 MHz for ^31^P NMR, and 100 MHz for ^13^C NMR) and processed with software Delta 5.0.5.

### Synthesis of Copper Oxide Nanoparticles

Copper(I) oxide (cuprous oxide, Cu_2_ONPs) and copper(II) oxide (cupric oxide, CuONSs) nanoparticles were prepared by chronoamperometry (at room temperature and at a constant electrode potential of 0.8 V for 4 h).^101^ A 0.1 M aqueous solution of lithium chloride (LiCl; from Sigma-Aldrich) was freshly prepared and used for CuONS synthesis, while an ethanolic LiCl solution with 10% water was freshly prepared and used for the synthesis of Cu_2_ONPs. The electrochemical treatment was carried out under an inert atmosphere by slowly bubbling the solution with argon gas in a conventional three-electrode cell with a platinum wire as a counter electrode and an Ag/AgCl (1 M KCl) electrode as a reference electrode (the potential is indicated against this electrode). A copper rod served as a working electrode. Before electrochemical treatment, metallic copper (99.99% Cu) was polished with sandpaper to reduce the grain size and then purified in anhydrous ethanol (99.8%; from Sigma-Aldrich). The precipitated product was in the form of orange Cu_2_ONPs and brown CuONSs.

### Ultraviolet–Visible (UV–Vis) Spectroscopy Measurements

UV–vis spectra of an aqueous copper oxide NSs sol and a sample/copper oxide NSs system measured after 180 min of mixing were recorded using a Lambda 25 UV–vis spectrometer.

### Scanning Electron Microscopy (SEM) Measurements

The SEM images of an aqueous copper oxide NSs sol were acquired using an SEM instrument, model S-5000 (Hitachi Ltd., Japan) at 20 kV.

### X-ray Powder Diffraction (XRD) Measurements

Powder X-ray diffraction (XRD) spectra were measured using a Rigaku UltimaIV X-ray diffractometer (Rigaku Co., Japan) with Cu K_α_ (λ = 1.542 Å) radiation at 40 kV and 40 mA in the range 20–80° (2θ) with a step of 0.02.

### Raman and Surface-Enhanced Raman Scattering (SERS) Measurements

Aqueous solutions of the studied compounds were prepared by dissolving each compound in deionized water (18 MΩ/cm; sample concentration 10^–4^ mol/dm^3^; pH = 7). A total of 10 μL of the sample solution was mixed with 20 μL of the aqueous sol solution. Then, 20 μL of the sample/sol mixture was applied to a glass plate and the SERS spectra were recorded (no measurements were made for the dried droplet). The spectra were recorded three times at three different locations on each surface.

The Raman and SERS spectra were recorded using a HoloSpec f/1.8i spectrograph (Kaiser Optical Systems Inc.) equipped with a liquid-nitrogen-cooled CCD detector (Princeton Instruments). The 785.0 nm line of a NIR diode laser (Invictus) was used as an excitation source. The laser power at the sample position was set to ∼15 mW. The typical exposure time for each SERS measurement was 40 s with four accumulations. The spectral resolution was set to 4 cm^–1^. The SERS spectra of a given adsorbate on a given substrate were almost identical, except for small differences (up to 5%) in some band intensities. No spectral changes that could be associated with decomposition of the sample were observed during these measurements.

The spectra obtained were almost identical (very well reproducible) except for small differences (up to ∼5%) in some band intensities. No spectral changes that could be associated with sample decomposition or desorption processes were observed in these measurements.

### Tip-Enhanced Raman Spectroscopy (TERS) Measurements

Aqueous sample solutions were prepared by dissolving the peptide in deionized water (18 MΩ/cm). The concentration of the sample was adjusted to 10^–4^ mol/dm^3^ before mixing with the colloidally suspended Cu_2_ONPs. Overall, 10 μL of the sample solution was mixed with 20 μL of Cu_2_ONPs. The mixture was kept for 15 min before SERS measurement. For TERS measurements, the mixture was placed on a glass plate after 15 min of incubation and dried in a vacuum dryer at 37 °C for 30 min.

TERS measurements were performed in a top-illumination top-collection setup using a 514 nm DPSS laser (Cobalt Fandago 25), a 90× objective (NA = 0.71), and a liquid-nitrogen-cooled CCD detector (Princeton Instrument). The tips used for the measurements were electrochemically etched bulk silver tips (Unisoku Co. Ltd.) with an apex radius of ∼50 nm, located 45° from the sample plane. The tips were attached to a 32 kHz tuning fork and were controlled by a noncontact AFM with shear force. The same tips were used for both AFM and TERS. For each measured point, spectra were first recorded under conditions where the tip approached the sample (the distance between the tip and the sample was less than 2 nm), and then under conditions where the tip retraced. Subtraction between the two conditions yielded a spectrum with the signal due to near-field enhancement (without the far-field signal from outside the tip). The typical exposure time for each SERS measurement was 240 s with 3 accumulations and a 0.10 mW laser power.

### Spectral Analysis

Spectral analysis was performed using a GRAMS/AI program (Galactic Industries Co., Salem, NH).

Multiple nonseparated bands were fitted using a GRAMS/AI program (Galactic Industries Co., Salem, NH). A 50/50% Lorentzian/Gaussian band shape was assumed and fixed for all bands.

## Supporting Information Available

General process for the preparation of PyPA; ^31^P, ^1^H, and ^13^C NMR spectra (in D_2_O) of compounds: 2-PyPA, 3-Py-PA, and 4-PyPA (PDF)
Molecular formula strings (CSV)

## Abbreviations Used

Cu_2_ONPs: copper(I) oxide nanoparticles
CuONSs: copper(II) oxide nanostructures
PyPA: analogues of α-aminophenylphosphinic acid of pyridine
SEM: scanning electron microscopy
SERS: surface-enhanced Raman scattering
TERS: tip-enhanced Raman scattering
UV–vis: ultraviolet–visible spectroscopy
XRD: X-ray diffraction analysis
