# Supplementary material for: SERS/TERS Characterization of New Potential Therapeutics: The Influence of Positional Isomerism, Interface Type, Oxidation State of Copper, and Incubation Time on Adsorption on the Surface of Copper(I) and (II) Oxide Nanoparticles
Source: J Med Chem. 2022 Mar 1;65(5):4387–400. doi: 10.1021/acs.jmedchem.2c00031 (PMC8919263; doi:10.1021/acs.jmedchem.2c00031)

Supporting Information

SERS/TERS characterization of new potential therapeutics: influence of positional isomerism, interface type, oxidation state of copper and incubation time on adsorption on the surface of copper(I) and (II) oxide nanoparticles

Edyta Proniewicz[a,b*] *and Tomasz K. Olszewski*[c]

[a] Faculty of Foundry Engineering, AGH University of Science and Technology, ul. Reymonta 23, 30-059 Kraków, Poland.

[b] Department of Chemistry, School of Science and Technology, Kwansei Gakuin University, Gakuen 2-1, Sanda, Hyogo 669-137, Japan.

[c] Faculty of Chemistry, Wroclaw University of Science and Technology, Wybrzeże Wyspiańskiego 27, 50-370 Wroclaw, Poland.

Corresponding Author email address: proniewi@agh.edu.pl

General process for the preparation of pyridine aminophosphinic acids

The synthesis protocol consisted of two successive reactions carried out in *one pot*. In the first step, the imines were prepared by reacting the corresponding pyridinecarboxaldehyde (1.07 g, 10 mMol) and benzylamine or butylamine (11 mMol) in methylene chloride (25 mL). The resulting mixture was stirred at room temperature for 24 hours and then anhydrous sodium

sulfate was added (to remove the water formed during the formation of the imine). After 20 minutes, the desiccant was removed by filtration and to the filtrate containing the crude imine was added to ethylphenylphosphinate (10 mMol) followed by bromotrimethylsilane (4.6 g, 30 mMol) (the second step). The resulting reaction mixture was stirred at room temperature for 24 hours. After this time, the mixture was evaporated to dryness in a vacuum rotary evaporator, forming oily products. Methanol (10 mL) was then added and the resulting mixture was placed in the refrigerator. The desired aminophosphinic acids precipitated as crystals and were collected by filtration, washed with cold diethyl ether (3x10 mL), and dried in air.

NMR spectra

Compound 2-PyPA: White solid. Yield 34%. M.p.: 179-180 ^oC. ^1H NMR (D_2O): δ (ppm) 8.30 (d, 1H, J = 4.8 Hz), 7.80 (t, 1H, J = 7.6 Hz), 7.40–7.27 (m, 7H), 4.61 (d, 1H, $J_{H\text{-}P}$ = 12.8 Hz), 3.03–2.88 (m, 2H), 1.59-1.48 (m, 2H), 1.27-1.15 (m, 2H), 0.70 (t, 3H, J = 7.2 Hz). ^{13}C NMR (D_2O): δ (ppm) 148.25, 140.30, 132.38, 132.00, 130.30, 128.62, 128.34, 125.19, 124.32, 63.25 (d, J = 82.9 Hz), 47.50, 27.15, 19.08, 12.07. ^{31}P NMR (D_2O): δ (ppm) 21.48 (s).

Compound 3-PyPA: White solid. Yield 51%. M.p.: 242-243 ^oC. ^1H NMR (D_2O): δ (ppm) 8.37-8.35 (m, 1H), 8.13 (s, 1H), 7.65 (m, 1H), 7.39–7.30 (m, 6H), 4.44 (d, 1H, $J_{H\text{-}P}$ = 11.6 Hz), 2.87-2.83 (m, 2H), 1.48-1.46 (m, 2H), 1.13-1.11 (m, 2H), 0.70 (t, 3H, J = 7.2 Hz). ^{13}C NMR (D_2O): δ (ppm) 148.77 (d, J = 5.2 Hz), 147.21, 137.33 (d, J = 4.0 Hz), 134.16, 132.90, 132.00, 131.91, 128.28 (d, J = 12.0 Hz), 123.80, 63.12 (d, J = 97.7 Hz), 47.69 (d, J = 10.9 Hz), 30.22, 19.64, 13.14. ^{31}P NMR (D_2O): δ (ppm) 22.29 (s).

Compound 4-PyPA: White solid. Yield 80%. M.p.: 171-173 ^oC. ^1H NMR (D_2O): δ (ppm) 8.45 (d, 2 H, J = 7.2 Hz), 7.55-7.53 (m, 2H), 7.38–7.14 (m, 10H), 4.66 (d, 1H, $J_{H\text{-}P}$ = 11.2 Hz), 4.30-4.21 (m, 2H). ^{13}C NMR (D_2O): δ (ppm) 152.57, 141.05, 132.97, 132.19, 132.10, 130.49, 130.18, 130.15, 129.34, 128.87, 128.75, 126.26, 126.23, 62.66 (d, J = 74.3 Hz), 52.08 (d, J = 4.6 Hz). ^{31}P NMR (D_2O): δ (ppm) 20.04 (s).

Compound 2-PyPA: ^{31}P NMR (D_2O)

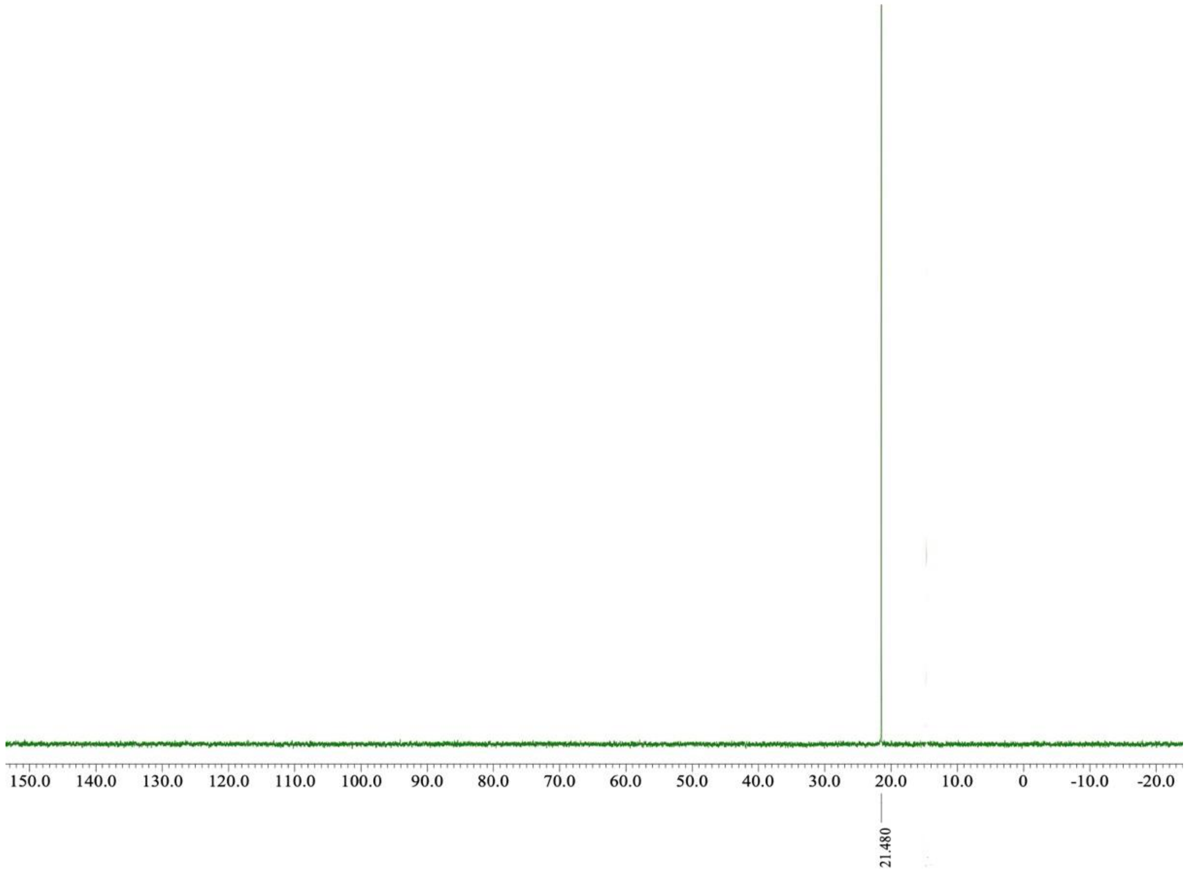

Compound 2-PyPA: ^{1}H NMR (D_2O)

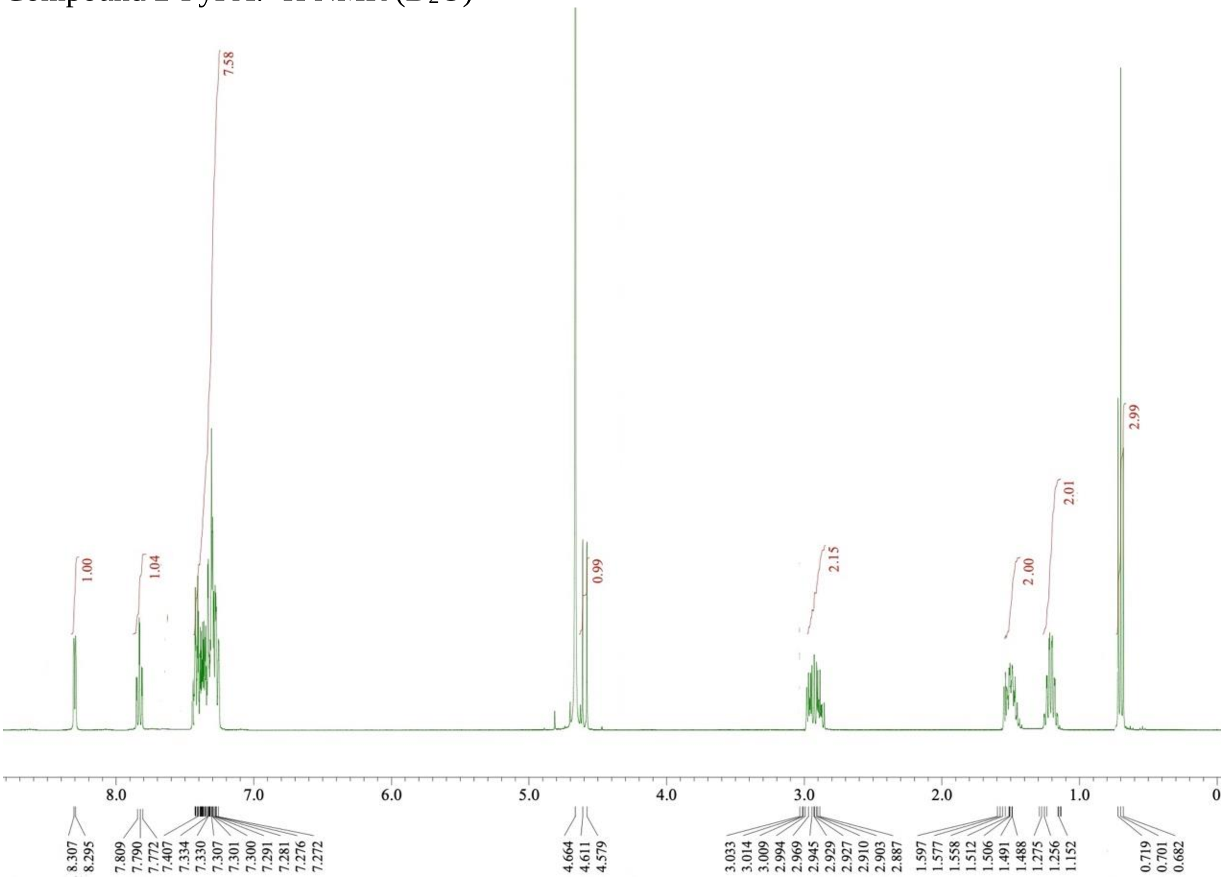

Compound 2-PyPA: ^{13}C NMR (D_2O)

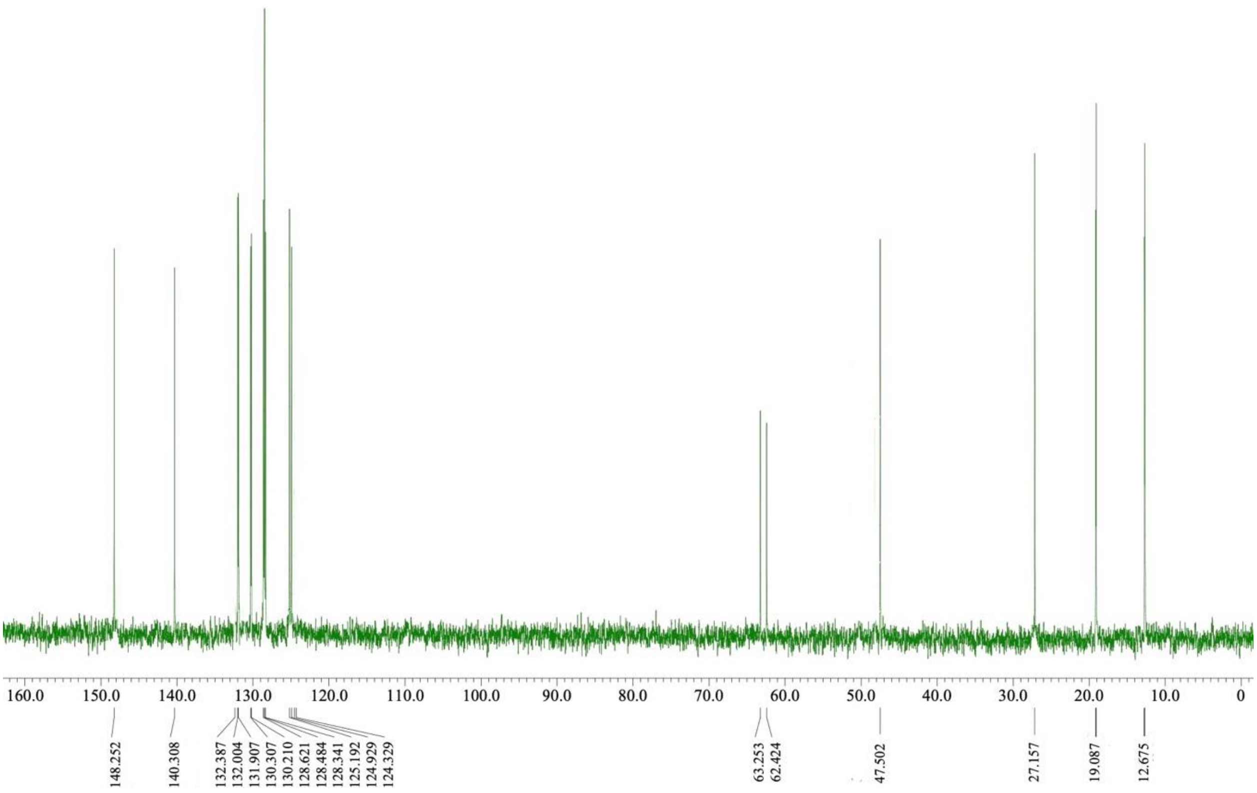

Compound 3-PyPA: ^{31}P NMR (D_2O)

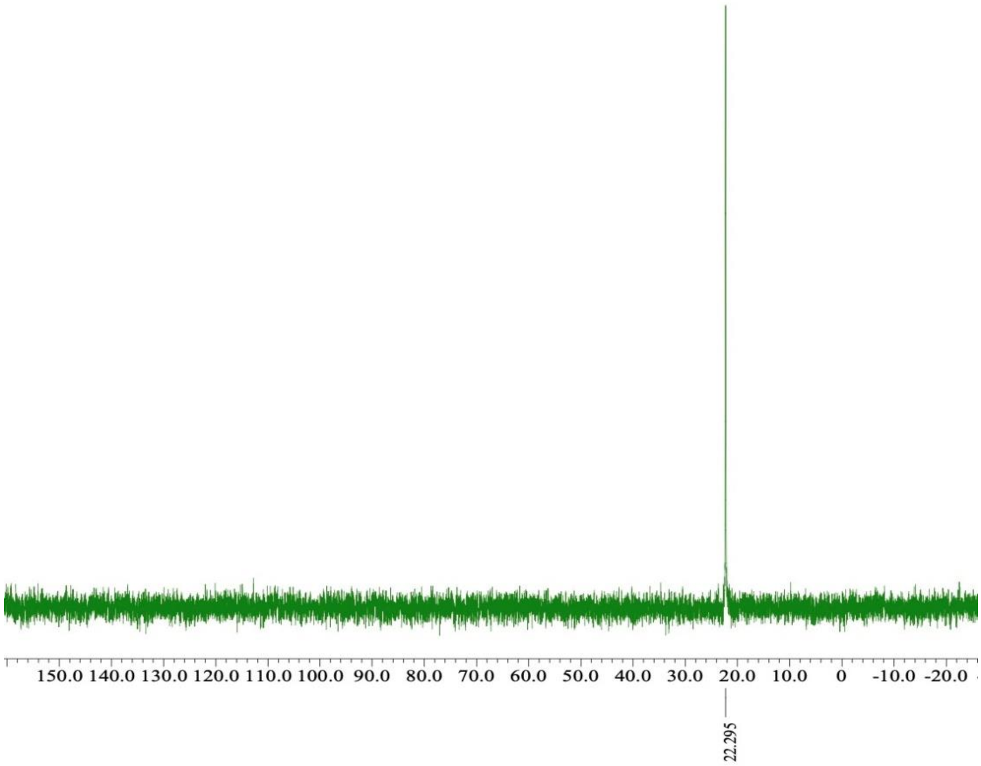

Compound 3-PyPA: ^{1}H NMR (D_2O)

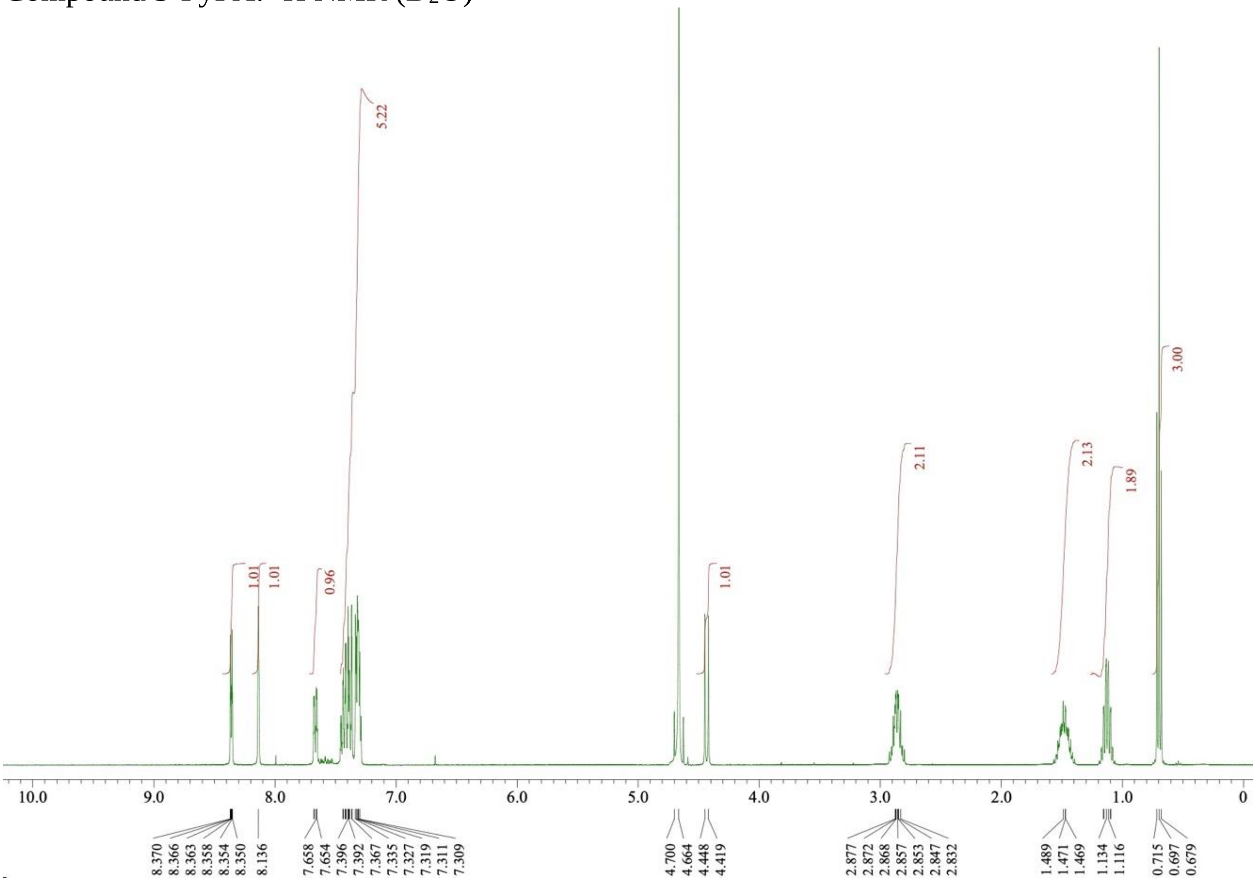

Compound 3-PyPA: ^{13}C NMR (D_2O)

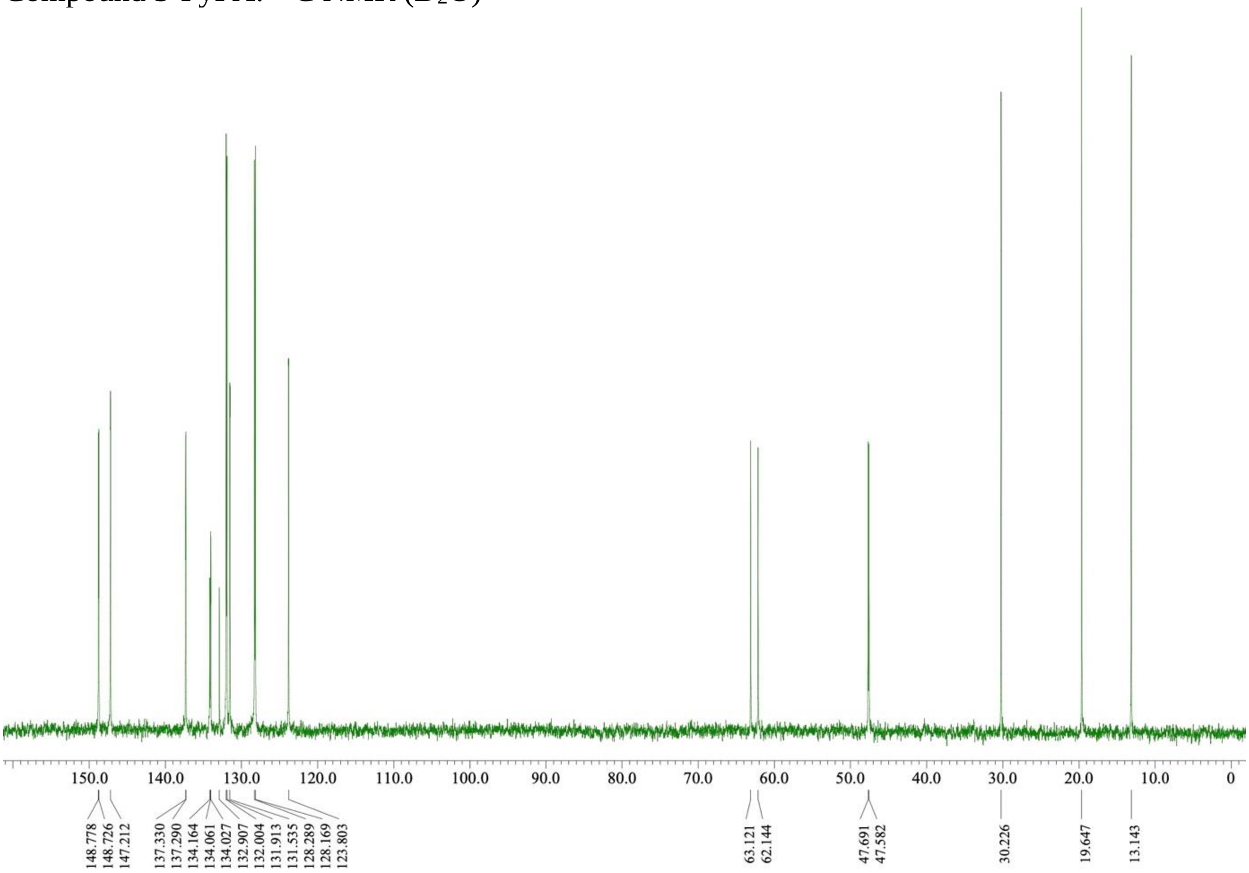

Compound 4-PyPA: ^{31}P NMR (D_2O)

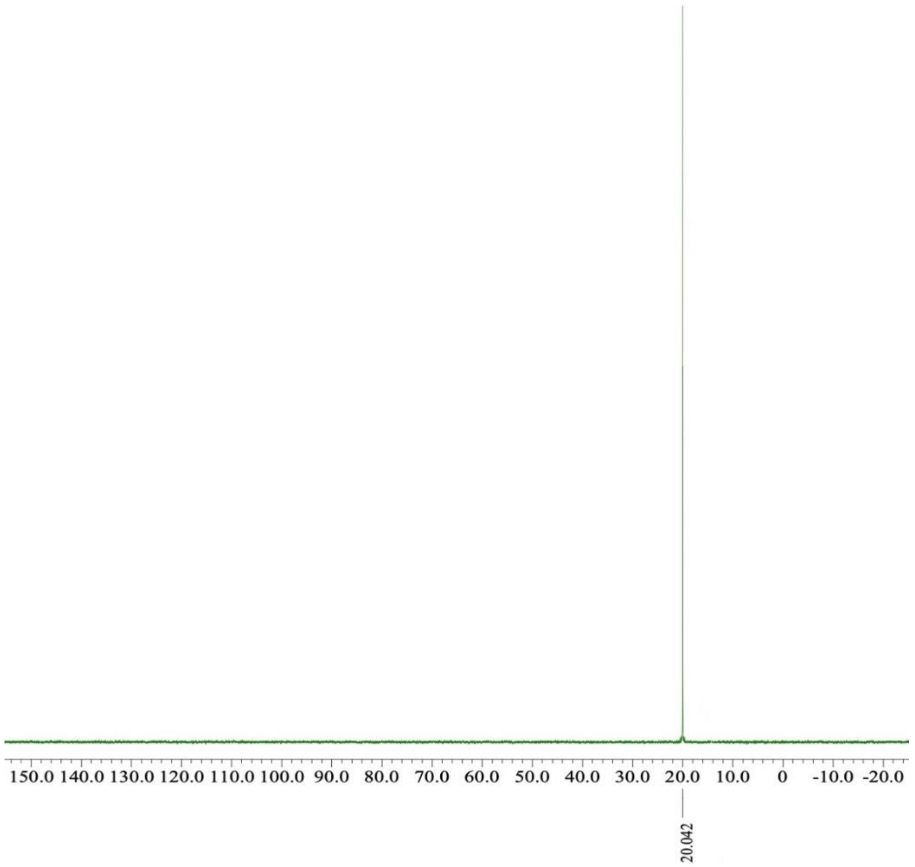

Compound 4-PyPA: ^{1}H NMR (D_2O)

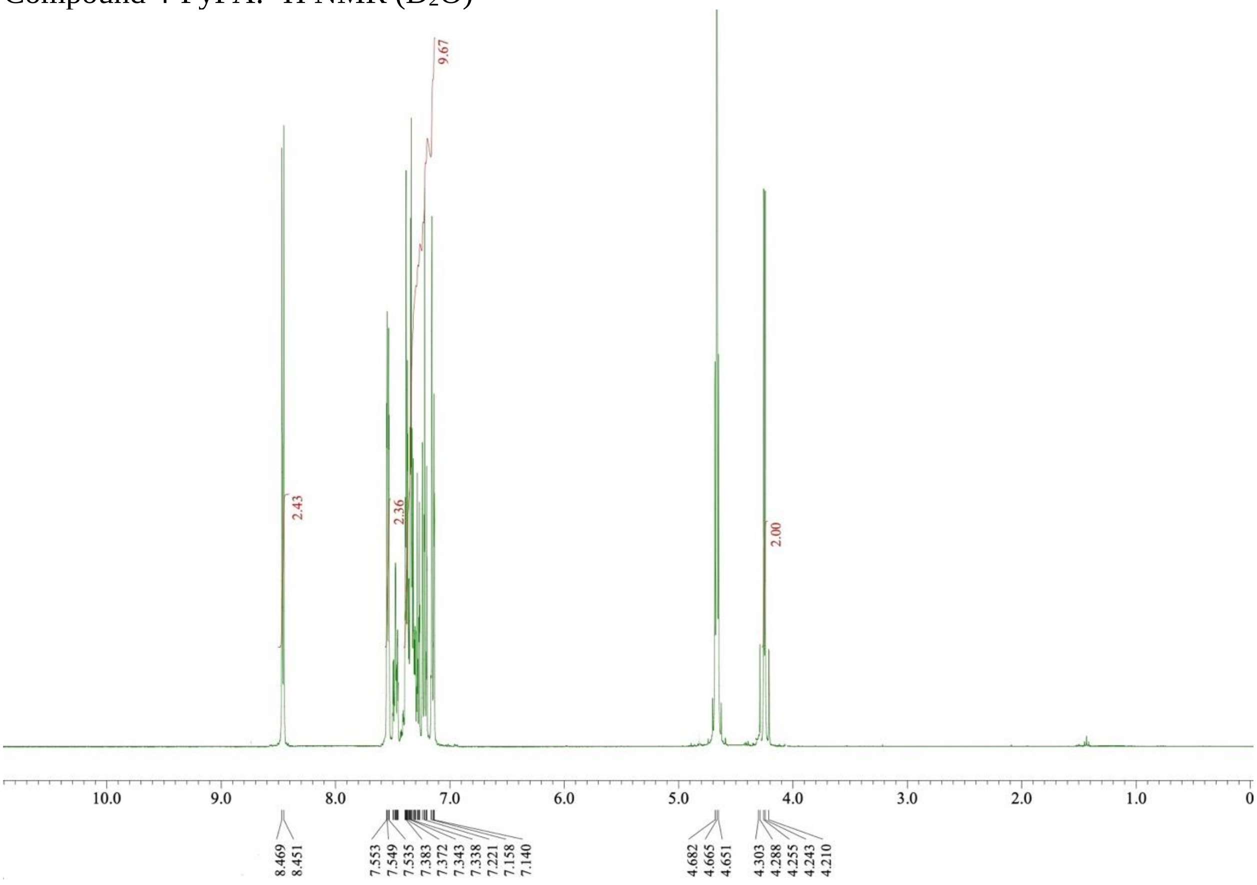

Compound 4-PyPA: ^{13}C NMR (D_2O)

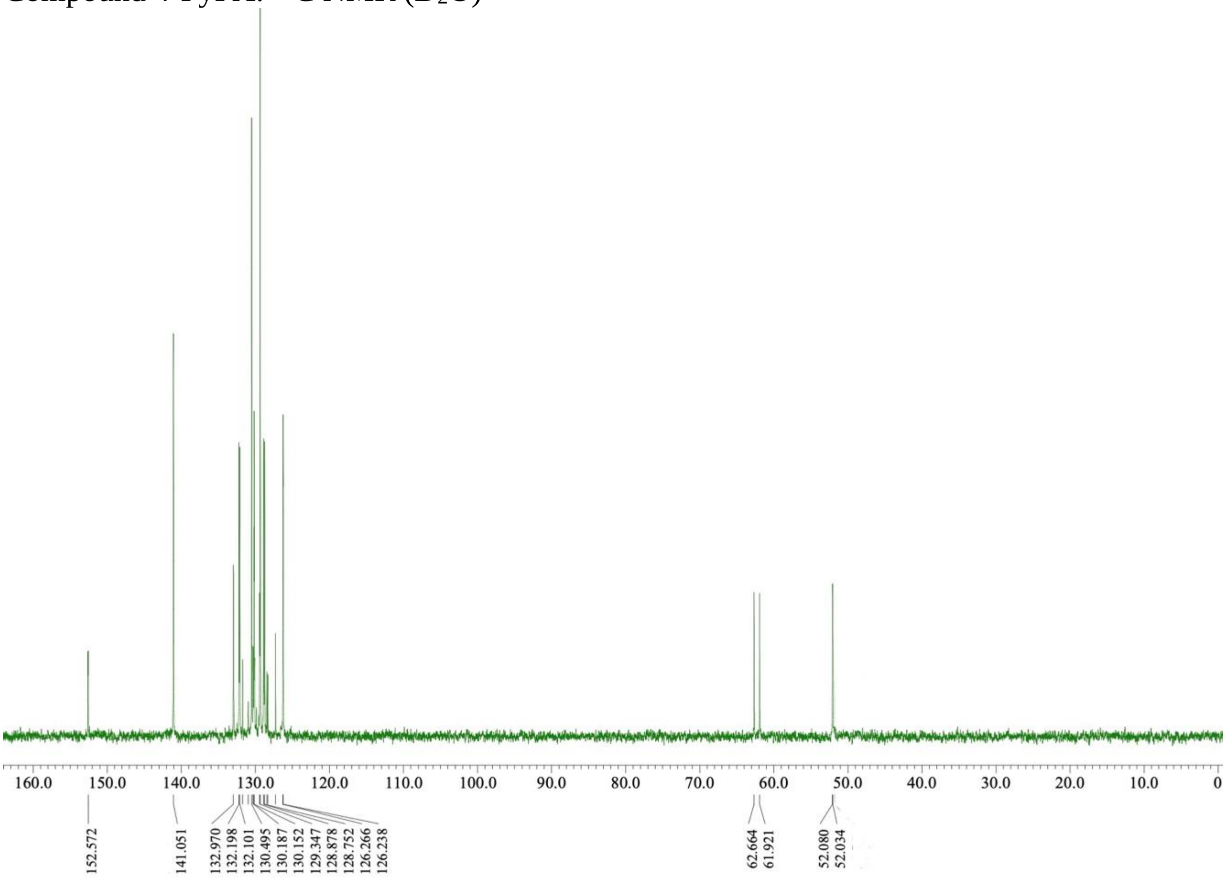

Supplement: Supplementary file 1 — jm2c00031_si_001.pdf [file jm2c00031_si_001.pdf]
